# Synthetic approach to iodosulfuron-methyl and metsulfuron-methyl metabolites and their application for water analysis†

**DOI:** 10.1039/d4ra01725k

**Published:** 2024-05-15

**Authors:** Marcin Rakowiecki, Sylwia Studzińska, Jacek Ścianowski, Mariusz J. Bosiak, Andrzej Wolan, Marcin Budny

**Affiliations:** a Synthex Technologies Sp. z o.o. Gagarina 7/134B 87-100 Toruń Poland budny@synthex.com.pl; b Department of Environmental Chemistry and Bioanalytics, Faculty of Chemistry, Nicolaus Copernicus University Gagarina 7 87-100 Toruń Poland; c Department of Organic Chemistry, Faculty of Chemistry, Nicolaus Copernicus University Gagarina 7 87-100 Toruń Poland; d Noctiluca S.A. Gagarina 7/41B 87-100 Toruń Poland

## Abstract

A synthetic approach to ten metabolites of iodosulfuron-methyl sodium and metsulfuron-methyl was performed and reported in this study. The compounds of interest were prepared by controlled hydrolytic degradation of active substances or by *de novo* synthesis from commercially available triazine precursor 10. Obtained compounds were characterized by IR, NMR, and elemental analysis techniques. Metabolites and active substances were utilized during the development of a separation and quantification method using reversed-phase high-performance liquid chromatography coupled with tandem mass spectrometry. The validated method was applied for the analysis of all studied compounds in the extracts from water samples collected from the Vistula river (Toruń, Poland).

## Abstract 2

Synthesis of iodosulfuron-methyl and metsulfuron-methyl metabolites and their application for water sample analysis from the Vistula river are reported.

## Graphical abstract

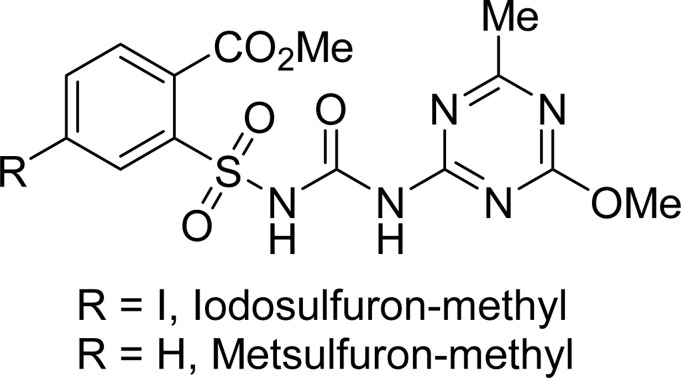

## Introduction

**Fig. 1:**
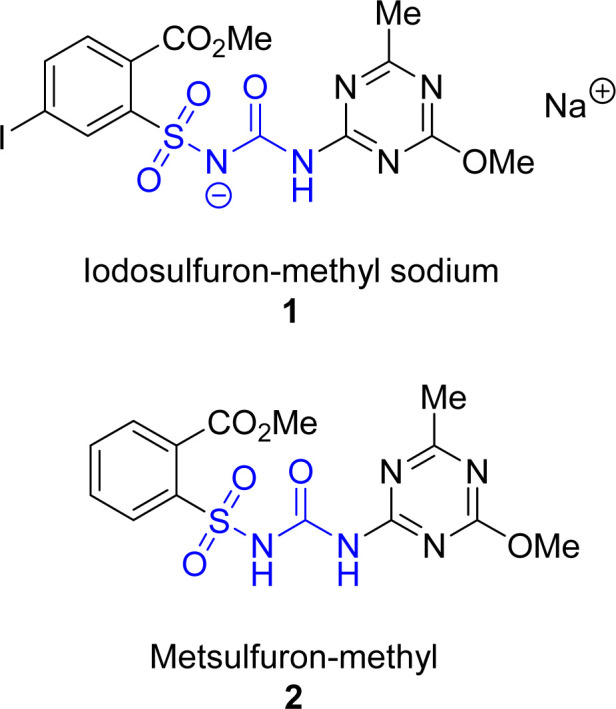
The structures of iodosulfuron-methyl sodium salt (1) and metsulfuron-methyl (2).

Iodosulfuron-methyl sodium (1)^1^ and metsulfuron-methyl (2)^2^ (Fig. 1), structurally related members of the sulfonylurea herbicide family, are widely used to control broadleaf weeds in plant cultivations.^3^ Their herbicidal properties come from inhibiting activity toward acetolactate synthase, an enzyme engaged in the synthesis of branched amino acids – l-leucine, l-isoleucine, and l-valine.^4^

Herbicides 1 and 2, like other sulfonylureas, undergo a number of processes providing compounds commonly referred to as metabolites when introduced to the environment. Some are plant, fungal, or bacterial metabolism products, and others are formed in hydrolytic and photochemical degradation reactions in soil and surface water.^5–13^

**Fig. 2:**
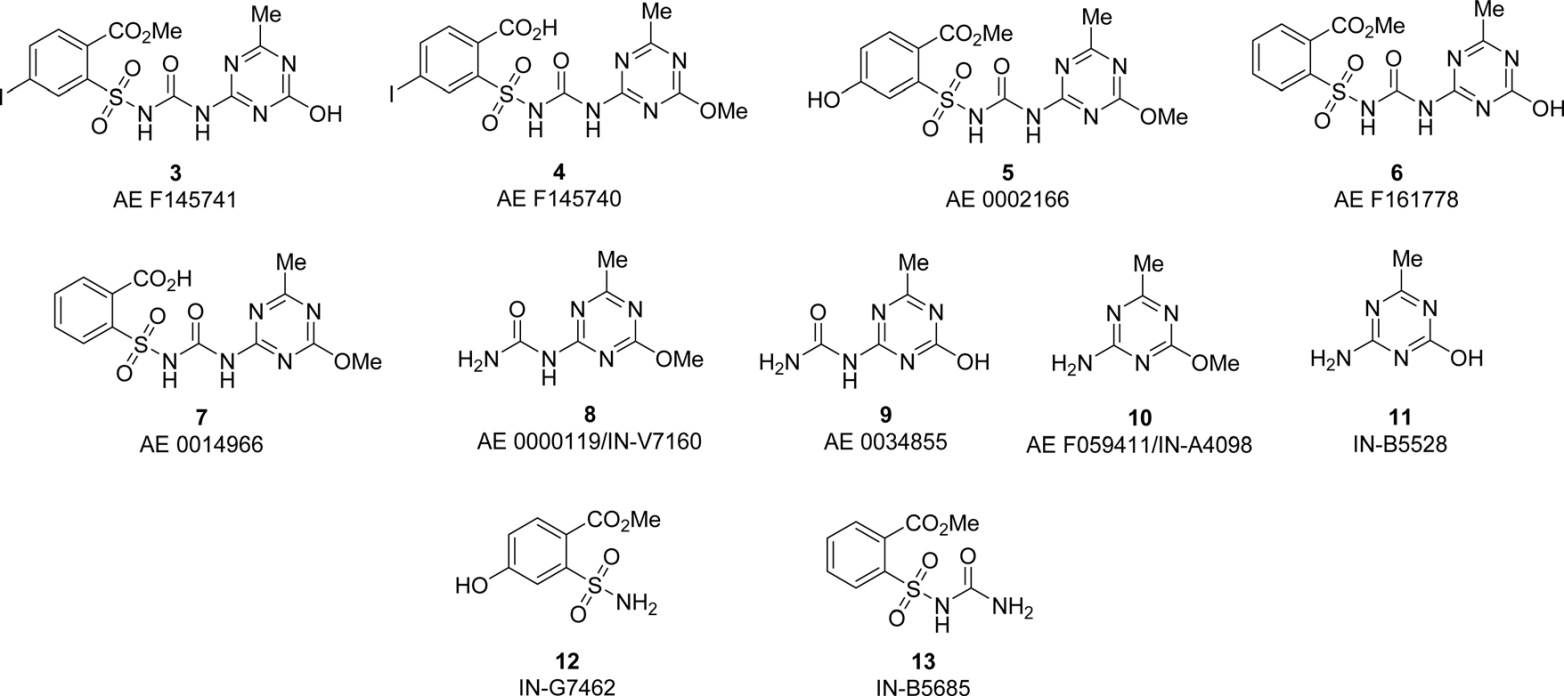
The structures of metabolites of iodosulfuron-methyl sodium (1) and metsulfuron-methyl (2) with accompanying codes reported in EFSA documentation.^1,2^

Experiments in which specific plant species were subjected to carbon-14 enriched herbicides allowed for identifying metabolites of 1 and 2.^14–19^ These results were also confirmed in controlled acid-base, photo- and biodegradation studies of nonlabelled active substances.^20–23^ The set of most relevant metabolites of 1 and 2, with corresponding codes reported in EFSA documentation, are summarized in Fig. 2. It should be noted that some of these structures are common for different sulfonylureas due to structural similarities between parent compounds.

One of our research interests was the development of synthetic methods that can be applied to the synthesis of agrochemicals' process impurities and metabolites.^24^ The properties of these compounds have to be extensively surveyed to fully assess the safety of active substances.^25^ Prompted by the commercial demand and recognizing that derivatives 3–13 are of limited availability, we decided to develop synthetic routes to these compounds. To show the utility of prepared compounds in environmental studies, we then used them as standards during the analysis of extracts from Vistula river water. For this purpose, solid-phase extraction was used for sample preparation, while reversed-phase high-performance liquid chromatography coupled with tandem mass spectrometry (RP HPLC MS/MS) was used for sample analysis.

## Results and discussion

### Synthesis of metabolites

**Scheme 1:**
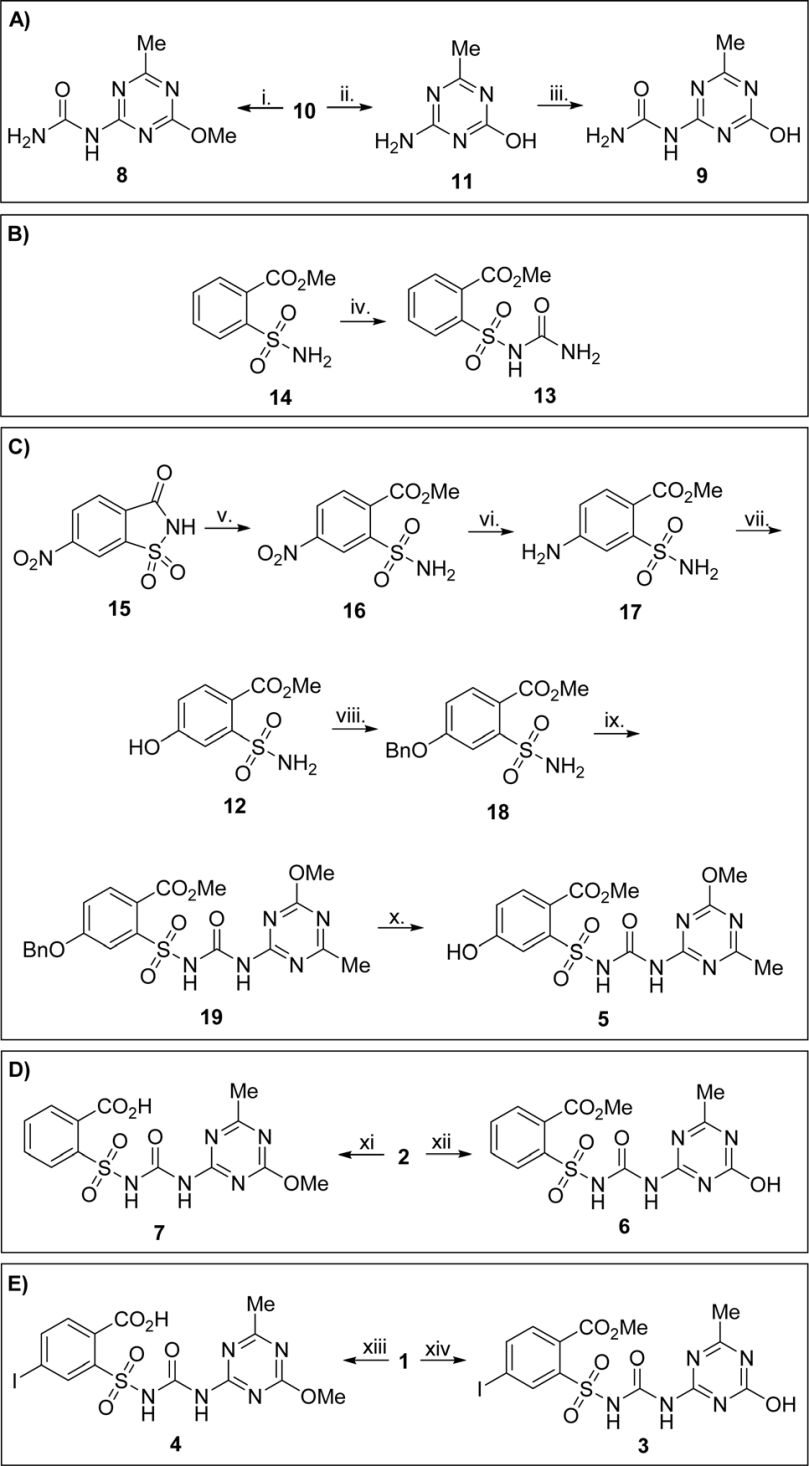
Synthesis of metabolites: (A) synthesis of ureas 8 and 9 (B) synthesis of sulfonylurea 13, (C) synthesis of sulfonylurea 5, (D) selective hydrolysis of metsulfuron-methyl (2), (E) selective hydrolysis of iodosulfuron-methyl sodium (1). Reagents and conditions: i. chlorosulfonyl isocyanate (1.5 equiv.), MeCN, 0 °C to rt, 1 h; then H_2_O, 0 °C, 1 h, 64%.; ii. NaOH (3.0 equiv.), H_2_O, 80 °C, 4 h; then 2 M HCl to pH 3, 85%.; iii. chlorosulfonyl isocyanate (1.5 equiv.), MeCN, 0 °C to rt, 1 h; then H_2_O, 0 °C, 1 h, 83%.; iv. chlorosulfonyl isocyanate (1 equiv.), 0 °C to rt, 1 h, 59%.; v. H_2_SO_4_ (0.66 equiv.), MeOH, reflux, 48 h, 94%.; vi. Pd/C, H_2_ (balloon), THF, overnight, 96%.; vii. NaNO_2_ (1.25 equiv.), H_2_SO_4_, H_2_O, 0 °C, 30 min; then urea (0.3 equiv.), 10 min; then Cu(NO_3_)_2_ (44.0 equiv.), Cu_2_O (2.0 equiv.), H_2_O, 88%.; viii. K_2_CO_3_ (1.2 equiv.), benzyl bromide (1.2 equiv.), acetone, reflux, 4 h, 54%.; ix. (COCl)_2_ (5.0 equiv.), DABCO (0.2 equiv.), toluene, 60 to 100 °C, overnight; then 10 (2 equiv.), toluene/EtOAc (1 : 1), pressure flask, 120 °C, 2 h, 62%.; x. Pd/C, H_2_ (balloon), EtOAc, 4 h, 55%., xi. *t*BuOK (2.0 eqiuv.), DMSO, rt, overnight; then 2 M HCl to pH 3, 65%.; xii. NaOH (2.0 equiv.), H_2_O, rt, 19 h; then 2 M HCl to pH 4, 63%.; xiii. *t*BuOK (1.75 equiv.), DMSO, rt, 30 min; then 12 M HCl to pH 4.5, 46%.; xiv. 12 M HCl, MeCN, 50 °C, 4 h, 49%.

Triazine-derived metabolites were synthesized from common precursor 10. Thus, the reaction of 10 with chlorosulfonyl isocyanate, followed by hydrolysis of sulfonyl intermediate, furnished urea 8 in moderate yield.^26^ Hydrolysis of 10 under basic conditions and subsequent carbamylation of 11 led to urea 9. Interestingly, the unprotected hydroxyl group in 11 did not interfere significantly with isocyanate reagent (Scheme 1A). Similarly, saccharin-derived sulfonamide 14 was transformed into corresponding urea 13 (Scheme 1B).

A longer synthetic sequence was required for obtaining metabolite 5. Tandem ring-opening/esterification of nitrosaccharin 15 yielded sulfonamide 16.^27^ Palladium-catalyzed hydrogenation of this compound, followed by diazotization/copper-mediated aqueous decomposition of diazonium salt, furnished phenol 12 in good overall yield. Its alkylation provided *O*-benzylated sulfonamide 18. Then, 18 was converted to corresponding isocyanate in a DABCO-catalyzed reaction,^28,29^ and this intermediate was directly subjected to reaction with 10, providing sulfonylurea 19 in 62% yield. Catalytic hydrogenation of 19 resulted in deprotection of the benzyl group and led to metabolite 5 (Scheme 1C).

Controlled hydrolysis of active substances 1 and 2 on a preparative scale was used to obtain metabolites 3, 4, 6, and 7 (Scheme 1D and E). *t*BuOK/DMSO system led to preferential hydrolysis of methyl ester in both cases, providing acids 4 and 7 in moderate yields. On the other hand, the methyl group in the triazine substituent was labile in aqueous NaOH or under acidic conditions, yielding 3 and 6, respectively. Structures of isomeric pairs of compounds 3/4 and 6/7 were unequivocally assigned through ESI MS fragmentation experiments performed in positive ionization mode. Thus, fragmentation of 3 and 6 provided hydroxytriazine cation *m*/*z* 153, whereas in cases 4 and 7, methoxytriazine cation *m*/*z* 168 was observed (Scheme S1, ESI†).

### Development of an analytical method

After establishing synthetic access to metabolites 3–13, we developed the RP HPLC-MS/MS analytical method and used these compounds as standards in the analysis of water samples.

For this purpose, full-scan mass spectra were recorded for each compound. The parent ions were then selected and fragmented to study the fragmentation paths and select product ions. Based on signals on MS/MS fragmentation spectra, MRM transitions were selected for all compounds, including parent herbicides 1 and 2 (Table S1, ESI†). In each case, two transitions were monitored (for the two most abundant ions at fragmentation spectra) for quantitative and qualitative analysis. Then, a mixture of standards was separated using the XB-C18 column in a MeOH/0.1% formic acid_(aq)_ mobile phase. The respective retention times of all compounds were presented in ESI (Table S1).† The separation took place in about 15 minutes. The chromatographic method was validated by the determination of basic parameters, which were presented in Table S2 (please see ESI for the details).† For all calibration curves, the coefficient of determination (*R*^2^) is equal or higher than 0.999, proving the linearity of the analytical method within the concentration range of 5 to 500 ng mL^−1^. Except for compounds 10 and 11, LOD and LOQ levels are 1.5–3.0 ng mL^−1^ and 5.0–10.0 ng mL^−1^, respectively. The differences may result from different ionization efficiencies for compounds 10 and 11 compared to others. Relative standard deviations for inter-day and intra-day precision parameters are less than 5% for all compounds (Table S2, ESI†).

### Extraction of compounds 1–13 from water and their determination

The solid phase extraction was used to isolate studied compounds from water samples. The non-polar C18 cartridge was applied since a similar type of adsorbent was used during the chromatographic analysis of compounds. First, the extraction method was optimized and developed for the standard solutions. The eluates from the SPE cartridge were collected at each procedure stage and analyzed by RP-HPLC MS/MS. This approach allowed us to check whether the compounds adsorb on the surface of the adsorbent, or they are not eluted at the washing stage, and how much is desorbed at the elution stage. All of the compounds were adsorbed at the surface of the SPE cartridge when their concentration was lower than 300 ng mL^−1^. Moreover, none of them were eluted from the adsorbent during the washing step.

Eluates from the SPE were analyzed by RP-HPLC MS/MS. Based on the results, the method is reliable and allows recovery of compounds 1–13 in the 79–105% range. Next, the developed SPE procedure was used for 500 mL of water sample collected from the Vistula river. The samples were concentrated 5000 times by the evaporation of SPE extract and reconstitution of residue in a small (100 µL) volume of MeOH. This step was necessary because, in environmental samples, these compounds occur at very low concentration levels. The results of RP-HPLC MS/MS analysis of extracts from the Vistula river are summarized in Table 1. Only active substances and metabolite 3 were identified and quantified. The levels of the remaining compounds were either below LOD or LOQ (Table S2, ESI†).

**Table 1:** The results of water samples analysis

Compound	Concentration [ng L^−1^]
1	0.4366
2	34.90
3	16.78

## Conclusions

In summary, a library of ten metabolites of 1 and 2 were synthesized. All these compounds, active substances, and commercial precursor 10 were used as chromatographic standards for RP-HPLC MS/MS water analysis. A suitable analytical method was developed and validated. The results confirmed the presence of three compounds in water samples, and the highest level was recorded for 2, while 11 was below LOQ.

## Experimental section

### General remarks

Acetonitrile (MeCN), ethyl acetate (EtOAc), and toluene were dried over activated 4 Å molecular sieves. Methanol (MeOH), 0.1% aqueous formic acid (HCO_2_H), and water used for RP HPLC-MS/MS analyses and sample preparations were purchased from J. T. Baker (VWR International, Poland) and were of LC-MS grade. Compounds 10 (AmBeed, USA), 14 (Aldrich, USA) and 15 (Fluorochem, UK) were commercially available and used without any further purification. Other solvents and reagents were of analytical grade and were used without further purification. Solid phase extraction C18 columns (1 g, 6 mL, Biotage, Sweden) and a glass vacuum chamber were used in solid phase extraction procedures. Syringe filters (PVDF, pore size 0.22 µm, CHEMLAND, Poland) were used for sample filtration. The Bruker Avance 300 MHz, 400 MHz, and 700 MHz spectrometer (Bruker, USA) were used to record nuclear magnetic resonance (NMR) spectra. Coupling constants are in hertz (Hz). The following abbreviations are used for spin multiplicity: s = singlet, d = doublet, t = triplet, q = quartet, m = multiplet, and br = broad. RP HPLC-MS/MS analyses were performed on a triple quadrupole Shimadzu LCMS 8030 (Shimadzu, Japan). Vario Macro CHN Element Analyzer (Elementar Analysen Systeme, Germany) was used for elemental analyses (C, H, N). Infrared spectra were recorded on a PerkinElmer UATR two instrument (PerkinElmer, Poland) and are reported in cm^−1^. Melting points were determined in open glass capillaries and were uncorrected. Silica Gel 60, Merck 230–400 (Merck, Germany), was used for preparative column chromatography. Sigma-Aldrich TLC plates (silica gel on Al foil with fluorescent indicator 254 nm, Aldrich, USA) were used for analytical TLC. UV lamp (*λ* = 254 nm).

### RP HPLC-MS/MS analysis

The qualitative and quantitative analyses were performed on a Shimadzu LCMS 8030 system. Electrospray ionization in positive mode was used in all analyses, except for compound 12, which was analyzed in negative ion mode since signal intensities were higher for this ionization mode.

The MS operating parameters are described in the following text. The nebulization gas flow was set up at 2.0 L min^−1^ and the dry gas flow rate at 15 L min^−1^. Capillary voltage was controlled by the tuning file, while the heating block temperature was equal to 400 °C, and the desolvation line temperature was 250 °C.

The RP HPLC operating parameters are described in the following text. A Phenomenex XB-C18 column (2.6 µm, 100 Å, 100 × 3.0 mm) was used to separate the mixture of compounds. Mobile phase flow rate was set up at 0.4 mL min; mobile phase consisted of 0.1% HCO_2_H(aq) and MeOH. The gradient elution was applied and program was as follows: (0–5 min. 3% MeOH, 5–15 min. 3–90% MeOH, 15–20 min. 90% MeOH). Column oven temperature was set up at 35 °C, while autosampler as 25 °C. Injection volume was set up as 1 µL.

### Chromatographic method validation

Seven standard solutions of all 13 compounds mixture were prepared from a stock solution to prepare calibration curves. The concentrations were in the range of 5–500 ng mL^−1^. The coefficient of determination (*R*^2^) was used to determine the linearity. A relative standard deviation of peak area for ten injections in one day for concentrations three different (25, 200, and 500 ng mL^−1^) was calculated to determine intra-day precision. To determine inter-day precision between different days of operation, a relative standard deviation of peak area for ten injections per day at three different concentrations (25, 200, and 500 ng mL^−1^) was calculated during the experiment's first, third, and seventh days. The limit of quantification (LOQ) was determined as a signal/noise ratio of more than 10. In contrast, the limit of detection (LOD) was determined as a signal/noise ratio of more than 3.

### Preparation of calibration solutions

A stock solution was prepared by dissolving samples of compounds 1–13 (5.0 mg each) in water to a volume of 100 mL using ultrasonic agitation. The concentration of each constituent is 0.05 mg mL^−1^.

Calibration solutions were prepared by diluting aliquots of stock solutions to a volume of 100 mL in water.

Final concentrations of each constituents 5 ng mL^−1^ (level 1), 10 ng mL^−1^ (level 2), 25 ng mL^−1^ (level 3), 50 ng mL^−1^ (level 4), 100 ng mL^−1^ (level 5), 200 ng mL^−1^ (level 6), 300 ng mL^−1^ (level 7), 400 ng mL^−1^ (level 8) and 500 ng mL^−1^ (level 9). Aliquots of calibration solutions were filtered through syringe filters before RP HPLC-MS/MS analyses (please see Table S2 in ESI† for details of calibration curves and other validation parameters).

### Extraction of studied compounds from standard solutions and water samples from Vistula river

The solid phase extraction was used for the isolation and purification of compounds from water samples. The following procedure was applied during the first step (extraction from the solution of a mixture of standards):

SPE cartridge: C18 (1 g, 6 mL).

Conditioning: 6 mL of MeOH, 6 mL of water.

Sample load: 3 mL of standard mixture at 50 and 300 ng mL^−1^ concentration level.

Washing: 6 mL of water.

Elution: 3 mL of MeOH.

Eluates from the SPE column were filtered through a syringe filter and subjected to RP HPLC MS/MS analysis.

Next, two water samples (A and B) from the Vistula river were collected on 31.01.2024 in Toruń, Poland (53°00′23.8″N, 18°35′56.5″E). Samples were collected about 1.5 meters from the shore using a non-metal scoop from a depth of about 50 cm. Water samples were collected into plastic bottles and immediately transported by car to the laboratory at a reduced temperature (in a polyurethane foam box filled with cooling blocks, internal temperature: 4 °C). The water samples were stored in a refrigerator (4 °C) before the SPE, but not longer than two hours. The time between sampling and SPE was not longer than three hours.

An attempt to extract studied compounds was made with the use of the developed SPE method. The 500 mL of water was passed through the SPE cartridge. The eluate was evaporated to dryness under a gentle stream of argon to enrich the sample due to the very low concentration of compounds in the environmental samples. The residue was reconstituted in MeOH (100 µL), filtered through a syringe filter, and subjected to RP HPLC MS/MS analysis.

### Procedures for the synthesis of metabolites

#### 1-(4-Methoxy-6-methyl-1,3,5-triazin-2-yl)urea (8)

To a solution of 10 (1 equiv., 21.41 mmol, 3.00 g) in anhydrous acetonitrile (30 mL), chlorosulfonyl isocyanate (1.5 equiv., 32.12 mmol, 4.50 g) was added at 0 °C under inert gas. The resulting mixture was stirred at ambient temperature for 1 h. Then, the reaction mixture was cooled to 0 °C, and water (6 mL) was added dropwise. Stirring was continued at room temperature for 1 h. During that time, the precipitate was formed, which was then filtered and washed with water (2 × 15 mL) and acetonitrile (15 mL). Solids were collected and air-dried at ambient temperature, affording 8 (2.53 g, 64% yield) as a white powder.

Mp. 277–279 °C; IR (neat, cm^−1^): 3318, 1681, 1545, 1340, 809; ^1^H NMR (700 MHz, DMSO-*d*_6_), *δ* (ppm): 9.95 (s, 1H), 8.27 (s, 1H), 7.31 (s, 1H), 3.91 (s, 3H), 2.40 (s, 3H).; ^13^C NMR (175 MHz, DMSO-*d*_6_), *δ* (ppm): 178.0, 170.2, 164.6, 153.8, 54.7, 25.1.; anal. calcd for C_6_H_9_N_5_O_2_: C, 39.34; H, 4.95; N, 38.23. Found: C, 39.36; H, 4.44; N, 38.23.

#### 4-Amino-6-methyl-1,3,5-triazin-2-ol (11)

To a solution of 10 (1.0 equiv., 71.38 mmol, 10.00 g) in water (60 mL), sodium hydroxide (3.0 equiv., 212.5 mmol, 8.50 g) was added. The resulting mixture was stirred at 80 °C for 4 hours, then cooled to 0 °C and acidified with 2 M hydrochloric acid to pH 3. The resulting precipitate was filtered and washed with water (2 × 50 mL). Solids were collected and air-dried at ambient temperature, affording 11 (7.70 g, 85% yield) as a white powder.

Mp. >333 °C; IR (neat, cm^−1^): 3088, 2941, 2824, 1597, 788, 669, 608; ^1^H NMR (700 MHz, D_2_O), *δ* (ppm): 2.12 (s, 3H). ^13^C NMR (100 MHz, D_2_O + NaOD), *δ* (ppm): 177.1, 170.6, 167.8, 23.4. Anal. calcd for C_4_H_6_N_4_O: C, 38.09; H, 4.80; N, 44.42. Found: C, 38.10; H, 4.74; N, 44.43.

#### 1-(4-Hydroxy-6-methyl-1,3,5-triazin-2-yl)urea (9)

The same procedure as for compound 8 was used. From compound 11 (1 equiv., 2.36 mmol, 1.0 g), compound 9 (0.89 g, 83% yield) was obtained as a pale yellow solid.

Mp. >300 °C (decomp.); IR (neat, cm^−1^): 1681, 1510, 1330, 800, 544; ^1^H NMR (700 MHz, DMSO-*d*_6_), *δ* (ppm): 12.30 (br s, 1H), 9.77 (s, 1H), 8.61 (s, 1H), 7.27 (s, 1H), 2.22 (s, 3H). ^13^C NMR (175 MHz, DMSO-*d*_6_), *δ* (ppm): 169.3, 163.9, 154.5, 154.0, 20.8.; anal. calcd for C_5_H_7_N_5_O_2_: C, 35.51; H, 4.17; N, 41.41. Found: C, 35.32; H, 4.11; N, 41.51.

#### Methyl 2-(*N*-carbamoylsulfamoyl)benzoate (13)

To a solution of 14 (1 equiv., 4.6 mmol, 1.00 g) in acetonitrile (10 mL), chlorosulfonyl isocyanate (1 equiv., 4.6 mmol, 0.65 g) was added at 0 °C. The resulting mixture was stirred for 1 h at ambient temperature. Then, solvents were removed under reduced pressure, and the solid residue was washed with water and filtered until the neutral pH of the filtrate. The solids were then washed with methanol (2 × 10 mL), diethyl ether (10 mL), and air-dried, affording 13 (0.71 g, 59% yield) as a white powder.

Mp. 172–174 °C; IR (neat, cm^−1^): 3405, 3292, 1117, 568, 558; ^1^H NMR (400 MHz, CDCl_3_), *δ* (ppm):8.17–8.12 (m, 1H), 7.87–7.81 (m, 1H), 7.67–7.59 (m, 2H), 5.70 (s, 2H), 3.98 (s, 3H). ^13^C NMR (100 MHz, DMSO-*d*_6_), *δ* (ppm): 167.2, 152.1, 137.5, 133.1, 131.7, 130.7, 130.3, 128.8, 53.0. Anal. calcd for C_9_H_10_N_2_O_5_S: C, 41.86; H, 3.90; N, 10.85. Found: C, 41.79; H, 3.85; N, 10.82.

#### Methyl 4-nitro-2-sulfamoylbenzoate (16)^30^

To a solution of 6-nitrosaccharin (15) (1 equiv., 329 mmol, 75.00 g) in methanol (700 mL), concentrated sulfuric acid (0.66 equiv., 216 mmol, 12 mL) was added. The resulting mixture was stirred at reflux for 48 h. Then, the reaction mixture was allowed to reach ambient temperature, and it was concentrated under reduced pressure to about 1/3 of its initial volume. Solids were collected, washed with cold methanol (100 mL), and air-dried affording 16 (80.1 g, 94% yield) as solid.

Mp. 191–193 °C; ^1^H NMR (700 MHz, DMSO-*d*_6_), *δ* (ppm): 8.73 (d, *J* = 2.3 Hz, 1H), 8.50 (dd, *J*_1_ = 8.3 Hz, *J*_2_ = 2.3 Hz, 1H), 7.94 (d, *J* = 8.3 Hz, 1H); 7.76 (s, 2H), 3.88 (s, 3H).

#### Methyl 4-amino-2-sulfamoylbenzoate (17)^30^

To a suspension of 10% Pd/C (2.80 g) in THF (600 mL), 16 (1 equiv., 230 mmol, 60.00 g) was added. The resulting mixture was degassed and then vigorously stirred at ambient temperature under positive hydrogen pressure overnight. After reaction completion, the catalyst was filtered off, and the solvent was removed under reduced pressure, furnishing 17 (51.06 g, 96% yield) as a pale yellow powder.

Mp. 183–184 °C; ^1^H NMR (700 MHz, DMSO-*d*_6_), *δ* (ppm): 7.60 (d, *J* = 8.5 Hz, 1H), 7.25 (d, *J* = 2.3 Hz, 1H); 7.06 (s, 2H), 6.69 (dd, *J*_1_ = 8.5 Hz, *J*_2_ = 2.3 Hz, 1H), 6.36 (br s, 2H), 3.78 (s, 3H).

#### Methyl 4-hydroxy-2-sulfamoylbenzoate (12)

To a solution of 17 (1.0 equiv., 222 mmol, 51.0 g) in 30% aqueous sulfuric acid (400 mL) at 0 °C, a solution of sodium nitrite (1.25 equiv., 277.5 mmol, 13.80 g) in water (155 mL) was added dropwise, at such a rate that the reaction mixture temperature did not rise above 5 °C. The resulting mixture was stirred at 0 °C for 30 min. Then, urea (0.3 equiv., 66.6 mmol, 4.00 g) was added, and stirring was continued for an additional 10 min. After that time, the reaction mixture was carefully poured into a solution of copper(ii) nitrate (44 equiv., 9.77 mol, 1833 g) in water (4000 mL). Copper(i) oxide (2 equiv., 443 mmol, 63.40 g) was added in portions to such solution, and the reaction mixture was stirred until the gas was released entirely. Sodium chloride (400 g) was added, and the product was extracted with EtOAc (3 × 300 mL). The combined organic layers were washed with water (4 × 400 mL), brine (400 mL) and dried with anh. MgSO_4_, filtered and concentrated under reduced pressure, affording 12 (45.10 g, 88% yield) as a light yellow powder.

Mp. 152–153 °C; IR (neat, cm^−1^): 3337, 3249, 1709, 1166, 692, 599, 506; ^1^H NMR (700 MHz, DMSO-*d*_6_), *δ* (ppm): 10.74 (s, 1H), 7.64 (d, *J* = 8.5 Hz, 1H), 7.41 (d, *J* = 2.4 Hz, 1H), 7.18 (s, 2H), 7.01 (dd, *J*_1_ = 8.5 Hz, *J*_2_ = 2.4 Hz 1H), 3.80 (s, 3H). ^13^C NMR (175 MHz, DMSO-*d*_6_), *δ* (ppm): 167.3, 160.0, 144.0, 132.4, 120.1, 118.0, 114.9, 52.6. Anal. calcd for C_8_H_9_NO_5_S: C, 41.56; H, 3.92; N, 6.06. Found: C, 41.69; H, 3.88; N, 6.12.

#### Methyl 4-(benzyloxy)-2-sulfamoylbenzoate (18)

To a solution of 12 (1 equiv., 195 mmol, 45.10 g) in acetone (300 mL), K_2_CO_3_ (1.2 equiv., 234 mmol, 32.30 g), and benzyl bromide (1.2 equiv., 234 mmol, 27.80 mL) were added. The resulting solution was stirred at reflux for 4 h. Then, the reaction mixture was cooled to ambient temperature, and solvents were removed under reduced pressure. The residue was washed with hexanes (2 × 300 mL) and Et_2_O (2 × 300 mL) and then suspended in EtOAc (300 mL), washed with water (2 × 300 mL) and brine (300 mL). The organic phase was dried over anh. MgSO_4_, filtered and concentrated under reduced pressure, affording 18 (34.00 g, 54% yield) as a white solid.

^1^H NMR (700 MHz, CDCl_3_), *δ* (ppm): 7.93 (d, *J* = 8.7 Hz, 1H), 7.79 (d, *J* = 2.6 Hz, 1H), 7.43–7.37 (m, 4H), 7.35–7.32 (m, 1H), 7.10 (dd, *J*_1_ = 8.6 Hz, *J*_2_ = 2.6 Hz, 1H), 5.88 (s, 2H), 5.16 (s, 2H), 3.95 (s, 3H). ^13^C NMR (100 MHz, CDCl_3_), *δ* (ppm): 167.4, 161.6, 144.2, 135.6, 133.7, 129.0, 128.7, 127.8, 121.3, 117.9, 115.5, 70.8, 53.3. Anal. calcd for C_13_H_13_NO_3_S: C, 59.30; H, 4.98; N, 5.32. Found: C,54.42H, 5.07; N, 5.24.

#### Methyl 4-(benzyloxy)-2-(*N*-((4-methoxy-6-methyl-1,3,5-triazin-2-yl)carbamoyl)sulfamoyl)benzoate (19)

To a suspension of 18 (1 equiv., 46.7 mmol, 15.0 g) in anh. toluene (550 mL), DABCO (0.2 equiv., 9.34 mmol, 1.05 g), and oxalyl chloride (5 equiv., 233 mmol, 20.43 mL) were added at ambient temperature. The reaction mixture was stirred at ambient temperature for 5 min, then at 60 °C for 6 h and finally at 100 °C overnight. Then, the reaction mixture was cooled to ambient temperature and concentrated under reduced pressure to about 1/3 of its initial volume. The residue was transferred to a pressure flask and a suspension of 10 (2 equiv., 93.4 mmol, 13.08 g) in an anh. toluene/EtOAc (1 : 1, 400 mL) was added. The pressure flask was tightly closed, and the reaction mixture was stirred at 120 °C for 2 h. Then, the reaction mixture was cooled to ambient temperature, and solvents were removed under reduced pressure. The residue was washed with diethyl ether (100 mL) and dissolved in EtOAc (600 mL). Insoluble solids were filtered off, whereas filtrate was concentrated under reduced pressure, affording 19 (14.09 g, 62%) as with solid.

^1^H NMR (700 MHz, CDCl_3_), *δ* (ppm): 8.00 (d, *J* = 2.6 Hz, 1H), 7.76 (d, *J* = 8.6 Hz, 1H), 7.45–7.35 (m, 5H), 7.17 (dd, *J*_1_ = 8.6 Hz, *J*_2_ = 2.6 Hz, 1H), 5.16 (s, 2H), 4.07 (s, 3H), 3.88 (s, 3H), 2.61 (s, 3H). ^13^C NMR (100 MHz, DMSO-*d*_6_), *δ* (ppm): 179.7, 170.9, 166.4, 163.6, 160.9, 148.7, 139.6, 135.6, 132.7, 128.9, 128.6, 127.9, 123.3, 119.6, 118.7, 71.0, 55.8, 52.9, 25.4. Anal. calcd. for C_19_H_19_N_5_O_5_S: C, 53.14; H, 4.46; N, 16.31. Found: C, 53.20; H, 5.54; N: 15.98.

#### Methyl 4-hydroxy-2-(*N*-((4-methoxy-6-methyl-1,3,5-triazin-2-yl)carbamoyl)sulfamoyl)benzoate (5)

To a suspension of 10% Pd/C (2.50 g) in EtOAc (800 mL), 18 (1 equiv., 28.7 mmol, 14.00 g) was added. The resulting mixture was degassed and then vigorously stirred at ambient temperature under positive hydrogen pressure for 4 h. After the reaction was completed, the catalyst was filtered off, and solvents were removed under reduced pressure. The residue was washed with hexanes (2 × 300 mL) and dissolved in Et_2_O (600 mL). Insoluble solids were filtered off, and the filtrate was washed with water (2 × 600 mL) and brine (600 mL) and dried over anh. MgSO_4_, filtered and concentrated under reduced pressure, affording 5 (6.26 g, 55% yield) as a white solid.

Mp. 174–176 °C; IR (neat, cm^−1^): 3398, 1721, 1167, 1120, 587; ^1^H NMR (700 MHz, CDCl_3_), *δ* (ppm): 7.91 (d, *J* = 2.6 Hz, 1H), 7.70 (d, *J* = 8.6 Hz, 1H), 7.06 (dd, *J*_1_ = 8.6 Hz, *J*_2_ = 2.6 Hz, 1H), 4.08 (s, 3H), 3.87 (s, 3H), 2.62 (s, 3H). ^13^C NMR (100 MHz, DMSO-*d*_6_), *δ* (ppm):178.4, 170.1, 166.2, 163.8, 159.6, 148.4, 138.4, 132.5, 121.2, 119.9, 118.5, 55.2, 52.6, 25.0.; anal. calcd for C_14_H_15_N_5_O_7_S: C, 42.32; H, 3.81; N, 17.62. Found: C, 42.35; H, 3.91; N, 17.67.

#### 2-(*N*-((4-Methoxy-6-methyl-1,3,5-triazin-2-yl)carbamoyl)sulfamoyl)benzoic acid (7)

To a solution of 2 (1 equiv., 0.026 mol, 10.00 g) in DMSO (70 mL), *t*BuOK (2 equiv., 0.052 mol, 5.83 g) was added. The resulting mixture was stirred at ambient temperature overnight; then, it was cooled in an ice-water bath and acidified with 2 M HCl to pH 3. The precipitate was filtered, washed with water (2 × 50 mL), and air-dried, affording 7 (6.26 g, 65% yield) as a white powder.

Mp. 158–160 °C; IR (neat, cm^−1^): 1685, 1234, 580, 516; ^1^H NMR (400 MHz, DMSO-*d*_6_), *δ* (ppm): 13.74 (br s, 1H), 12.44 (s, 1H), 11.04 (s, 1H), 8.20–8.12 (m, 1H), 7.86–7.73 (m, 3H), 3.99 (s, 3H), 2.48 (s, 3H). ^13^C NMR (100 MHz, DMSO-*d*_6_), *δ* (ppm): 178.5, 170.1, 168.0, 163.8, 148.5, 135.6, 134.1, 133.3, 131.2, 130.6, 129.6, 55.2, 25.1. Anal. calcd for C_13_H_13_N_5_O_6_S: C, 42.51; H, 3.57; N, 19.07. Found: C, 42.53; H, 3.49; N, 19.15.

#### Methyl 2-(*N*-((4-hydroxy-6-methyl-1,3,5-triazin-2-yl)carbamoyl)sulfamoyl)benzoate (6)

To a suspension of 2 (1 equiv., 0.20 mol, 76.27 g) in water (700 mL), NaOH (2 equiv., 0.40 mol, 16.00 g) was added, and the resulting mixture was stirred at ambient temperature for 19 h. Then, the reaction mixture was diluted with water (200 mL), solids were filtered off, and the filtrate was acidified with 2 M HCl to pH 4. The resulting precipitate was filtered, washed with water (4 × 400 mL), and air-dried. The crude product was suspended in DCM (400 mL) and stirred at ambient temperature for 0.5 h. Then, solids were collected by filtration, washed with an additional portion of DCM (140 mL), and air-dried, affording 6 (46.21 g, 63% yield) as a white powder.

Mp. 194 °C (decomp.); IR (neat, cm^−1^): 2960, 2841, 1683, 581, 569; ^1^H NMR (400 MHz, DMSO-*d*_6_), *δ* (ppm): 13.05 (s, 2H), 11.00 (s, 1H), 8.17–8.14 (m, 1H), 7.83–7.79 (m, 2H), 7.73–7.71 (m, 1H), 3.88 (s, 3H), 2.26 (s, 3H). ^13^C NMR (100 MHz, DMSO-*d*_6_), *δ* (ppm): 171.1, 167.1, 163.2, 153.0, 149.2, 136.0, 134.1, 132.0, 131.2, 130.9, 129.5, 53.2, 21.1.; anal. calcd for C_13_H_13_N_5_O_6_S: C, 42.51; H, 3.57; N, 19.07. Found: C, 42.67; H, 3.59; N, 19.13.

#### 4-Iodo-2-(*N*-((4-methoxy-6-methyl-1,3,5-triazin-2-yl)carbamoyl)sulfamoyl)benzoic acid (4)

To a solution of 1 (1 equiv., 2.00 mmol, 1.01 g) in DMSO (10 mL), *t*BuOK (1.75 equiv., 3.5 mmol, 0.40 g) was added, and the resulting mixture was stirred at ambient temperature for 30 min. Then, the reaction mixture was diluted with water (20 mL), cooled in an ice-water bath to 0 °C, and acidified with 12 M HCl to pH 4.5. Solids were collected by filtration, then were washed with water (2 × 10 mL), 2-propanol (2 × 10 mL), Et_2_O (2 × 10 mL), air-dried affording 4 (0.45 g, 46% yield) a white powder.

Mp. 148–150 °C; IR (neat, cm^−1^): 3321, 3188, 1155, 586, 572; ^1^H NMR (700 MHz, DMSO-*d*_6_), *δ* (ppm): 13.75 (br s, 1H), 12.53 (br s, 1H), 11.11 (s, 1H), 8.38 (d, *J* = 1.7 Hz, 1H), 8.19 (dd, *J*_1_ = 8.2 Hz, *J*_2_ = 1.7 Hz, 1H), 7.54 (d, *J* = 8.2 Hz, 1H), 3.98 (s, 3H), 2.47 (s, 3H). ^13^C NMR (175 MHz, DMSO-*d*_6_), *δ* (ppm): 178.4, 170.1, 167.4, 163.8, 148.5, 142.7, 139.0, 136.8, 132.5, 131.3, 96.9, 55.2, 25.1. Anal. calcd for C_13_H_12_IN_5_O_6_S: C, 31.66; H, 2.45; N, 14.20. Found: C, 31.79; H, 2.45; N, 14.24.

#### Methyl 2-(*N*-((4-hydroxy-6-methyl-1,3,5-triazin-2-yl)carbamoyl)sulfamoyl)-4-iodobenzoate (3)

To a solution 1 (1 equiv., 39.00 mmol, 20.00 g) in MeCN (200 mL), 12 M HCl (6.15 equiv., 0.24 mol, 20 mL) was added. The resulting mixture was stirred at 50 °C for 4 h. Then, the reaction mixture was cooled to ambient temperature, and solids were collected by filtration. The solids were then washed with MeCN (100 mL), water (2 × 100 mL), and MeOH (100 mL) and air-dried, affording 3 (9.53 g, 49% yield) as a white powder.

Mp. 198–200 °C; IR (neat, cm^−1^): 3301, 1704, 1198, 1165, 583; ^1^H NMR (700 MHz, DMSO-*d*_6_), *δ* (ppm): 13.25 (br s, 1H), 11.15 (s, 1H), 8.38 (d, *J* = 1.6 Hz, 1H), 8.18 (dd, *J*_1_ = 8.0 Hz, *J*_2_ = 1.6 Hz, 1H), 7.48 (d, *J* = 8.0 Hz, 1H), 3.88 (s, 3H), 2.29 (s, 3H). ^13^C NMR (175 MHz, DMSO-*d*_6_), *δ* (ppm): 176.3, 170.1, 163.1, 162.6, 157.8, 141.1, 140.6, 136.7, 130.2, 129.6, 96.2, 53.8, 23.1.; anal. calcd for C_13_H_12_IN_5_O_6_S: C, 31.66; H, 2.45; N, 14.20. Found: C, 31.92; H, 2.35; N, 14.09.

## Conflicts of interest

There are no conflicts to declare.

## Supplementary Material

RA-014-D4RA01725K-s001
