# Supplementary material for: Synthetic approach to iodosulfuron-methyl and metsulfuron-methyl metabolites and their application for water analysis
Source: RSC Adv. 2024 May 15;14(23):15848–55. doi: 10.1039/d4ra01725k (PMC11095237; doi:10.1039/d4ra01725k)
Supplement: RA-014-D4RA01725K-s001 [file RA-014-D4RA01725K-s001.pdf]

Electronic Supplementary Material (ESI) for RSC Advances.
This journal is © The Royal Society of Chemistry 2024

Electronic supporting information for:

Synthetic approach to iodosulfuron-methyl and metsulfuron-methyl metabolites and their application for water analysis

Marcin Rakowiecki[a], Sylwia Studzińska[b], Jacek Ścianowski[b], Mariusz J. Bosiak[c,d], Andrzej Wolan[a,b], Marcin Budny*[,a]

[a] Synthex Technologies Sp. z o.o., Gagarina 7/134B, 87-100 Toruń, Poland. [b] Department of Environmental Chemistry and Bioanalytics, Faculty of Chemistry, Nicolaus Copernicus University, Gagarina 7, 87-100 Toruń, Poland. [c] Department of Organic Chemistry, Faculty of Chemistry, Nicolaus Copernicus University, Gagarina 7, 87-100 Toruń, Poland, [d] Noctiluca S.A., Gagarina 7/41B, 87-100 Toruń, Poland.

Table of contents

Scheme S1. Plausible MS/MS fragmentations of **3**/**4** and **6**/**7**......2

Figure S1. ESI MS/MS chromatograms of compounds **1**-**13**......3

Table S1. Retention times and MRM conditions for compounds **1**-**13**......4

Table S2. Analytical parameters for determination of compounds **1**-**13**......5

^{1}H NMR and ^{13}C NMR spectra......6

Scheme S1. Plausible MS/MS fragmentations of **3/4** and **6/7**

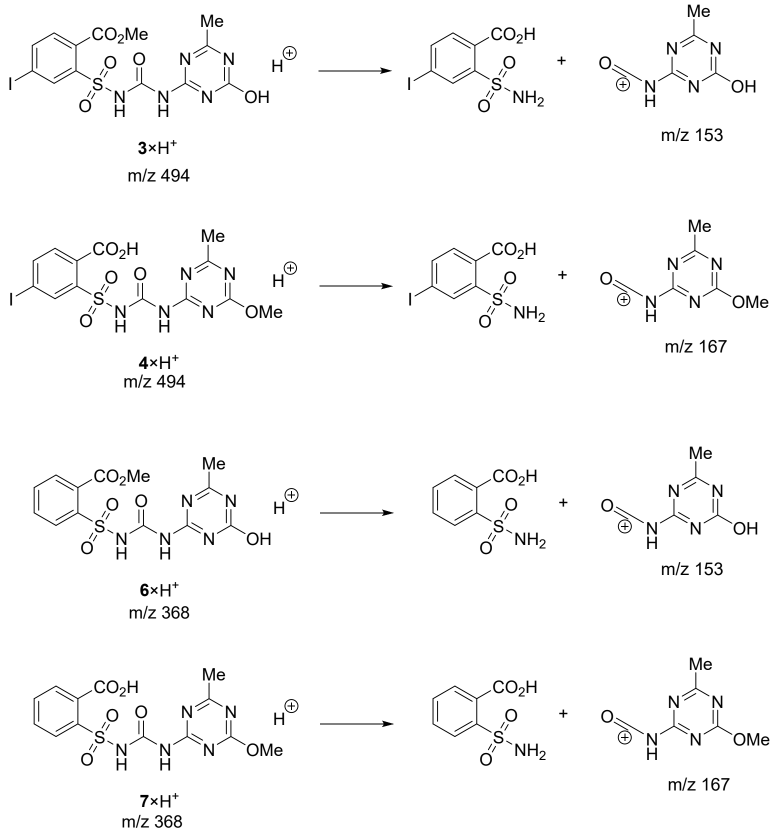

Figure S1. ESI MS/MS chromatograms of compounds **1**-**13**

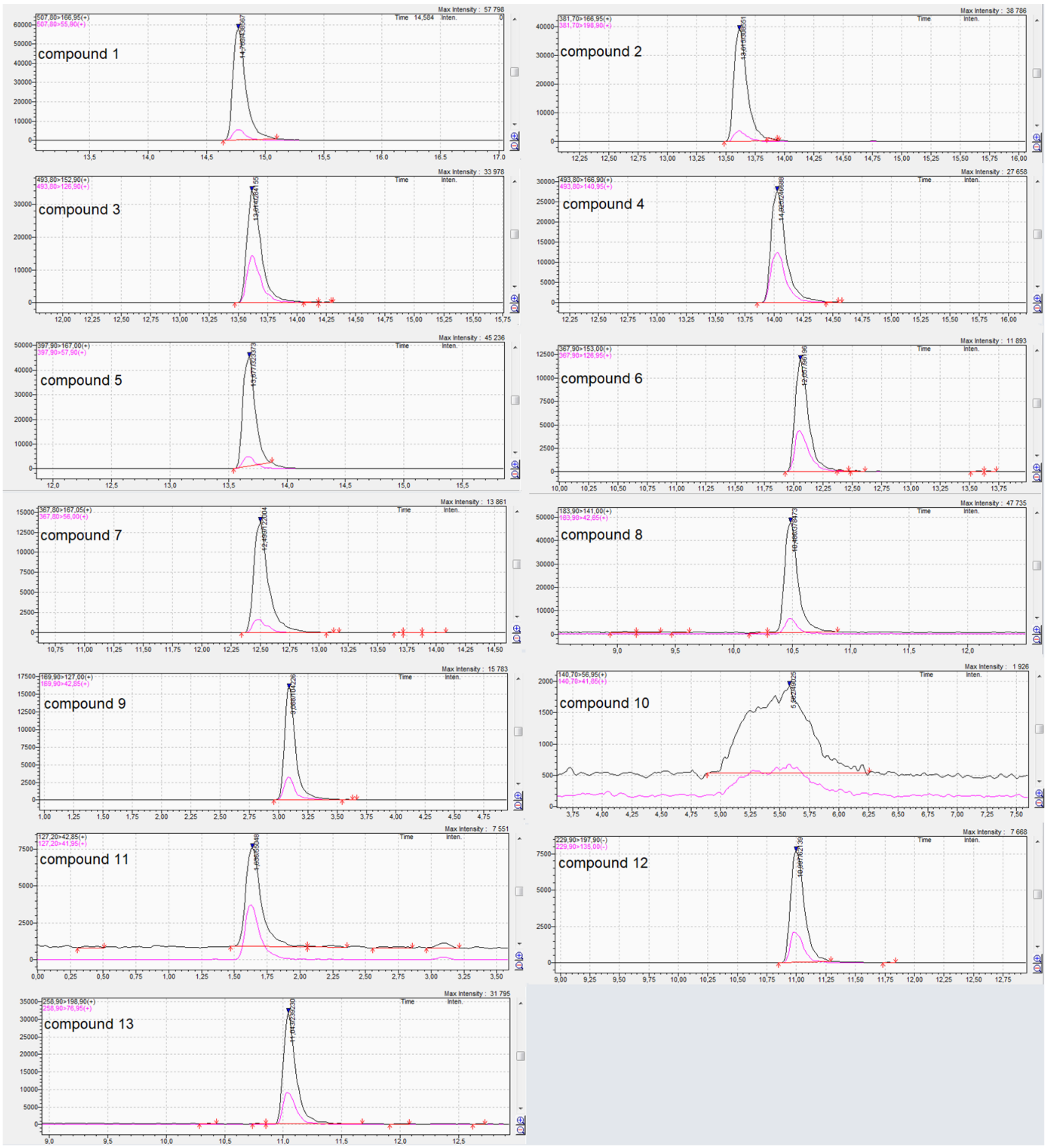

Table S1. Retention times and MRM conditions for compounds **1-13**.

Compound	Retention time [min]	Precursor ion	Product ions	Dwell time [ms]	Q1 [V]	CE	Q3 [V]
**1**	14.90	507.80	166.95	56.0	-26.0	-25.0	-28.0
			55.90	56.0	-26.0	-55.0	-20.0
**2**	13.69	381.70	166.95	37.0	-19.0	-19.0	-30.0
			198.90	37.0	-19.0	-22.0	-17.0
**3**	13.67	493.80	152.90	37.0	-24.0	-21.0	-25.0
			126.90	37.0	-24.0	-24.0	-20.0
**4**	14.10	493.80	166.90	37.0	-24.0	-22.0	-29.0
			140.95	37.0	-24.0	-28.0	-22.0
**5**	13.73	397.90	167.00	37.0	-30.0	-18.0	-29.0
			57.90	37.0	-30.0	-54.0	-21.0
**6**	12.06	367.90	153.00	37.0	-29.0	-14.0	-26.0
			126.95	37.0	-29.0	-17.0	-21.0
**7**	12.51	367.80	167.05	37.0	-26.0	-18.0	-30.0
			58.00	37.0	-26.0	-41.0	-20.0
**8**	10.54	183.90	141.00	37.0	-21.0	-13.0	-24.0
			42.85	37.0	-21.0	-31.0	-16.0
**9**	3.96	169.90	127.00	264.0	-18.0	-12.0	-20.0
			42.85	264.0	-18.0	-34.0	-13.0
**10**	6.78	140.70	56.95	264.0	-30.0	-19.0	-20.0
			41.85	264.0	-30.0	-25.0	-13.0
**11**	1.74	127.20	42.85	264.0	-13.0	-16.0	-15.0
			41.95	264.0	-13.0	-25.0	-14.0
**12**[a)]	11.02	229.90	197.90	37.0	+17.0	+14.0	+19.0
			135.00	37.0	+17.0	+22.0	+23.0
**13**	11.06	258.90	198.90	37.0	-28.0	-9.0	-19.0
			76.95	37.0	-28.0	-38.0	-29.0

a) The MRM performed in negative mode.

Table S2. Analytical parameters for determination of compounds **1-13**.

Compound	Regression equation	R^2	LOD [ng/mL]	LOQ [ng/mL]	RSD (%) Intra-day precision 25 [ng/mL]	RSD (%) Intra-day precision 200 [ng/mL]	RSD (%) Intra-day precision 500 [ng/mL]	RSD (%) Inter-day precision 25 [ng/mL]	RSD (%) Inter-day precision 200 [ng/mL]	RSD (%) Inter-day precision 500 [ng/mL]	Recovery [%] 50 [ng/mL]	Recovery [%] 300 [ng/mL]
**1**	y = 994913x + 5304.8	0.9991	1.5	5.0	3.7	2.8	3.4	2.2	3.0	2.6	99 ± 5	101 ± 3
**2**	y = 501575x + 4875.9	0.9997	3.0	10.0	4.2	5.2	4.1	2.1	4.4	3.2	105 ± 1	103 ± 1
**3**	y = 575947x + 1201.2	0.9997	3.0	10.0	4.0	3.3	3.2	4.2	2.0	3.1	94 ± 4	105 ± 3
**4**	y = 502142x + 1677.8	0.9992	1.5	5.0	4.1	2.5	4.4	5.2	4.1	4.6	83 ± 5	91 ± 2
**5**	y = 549107x + 3335.7	0.9993	1.5	5.0	2.5	1.2	3.6	2.8	2.9	2.9	93 ± 4	101 ± 5
**6**	y = 292160x + 1085.7	0.9989	3.0	10.0	5.5	1.6	2.1	4.0	4.0	3.0	99 ± 2	102 ± 5
**7**	y = 343333x + 1311	0.9993	1.5	5.0	4.2	2.4	2.6	4.5	1.8	4.1	92 ± 3	79 ± 5
**8**	y = 845574x + 4800.4	0.9990	3.0	10.0	3.9	1.1	2.5	4.1	1.9	3.0	102 ± 4	103 ± 4
**9**	y = 269056x - 406.02	0.9999	3.0	10.0	4.1	3.9	3.9	3.2	3.4	4.8	101 ± 3	105 ± 1
**10**	y = 160621x + 843.99	0.9997	7.5	25.0	2.2	2.9	2.6	4.9	3.9	2.8	104 ± 3	105± 2
**11**	y = 94693x + 76.7	0.9996	60.0	200.0	nd.	4.0	4.9	nd.	4.8	5.0	102 ± 5	104 ± 4
**12**	y = 256865x + 5107.5	0.9989	3.0	10.0	1.2	4.8	2.6	4.5	4.5	4.5	105 ± 4	102 ± 4
**13**	y = 637448x + 2656.2	0.9990	1.5	5.0	4.6	2.2	2.2	4.1	3.3	3.7	98 ± 4	80 ± 5

^{1}H NMR and ^{13}C NMR spectra

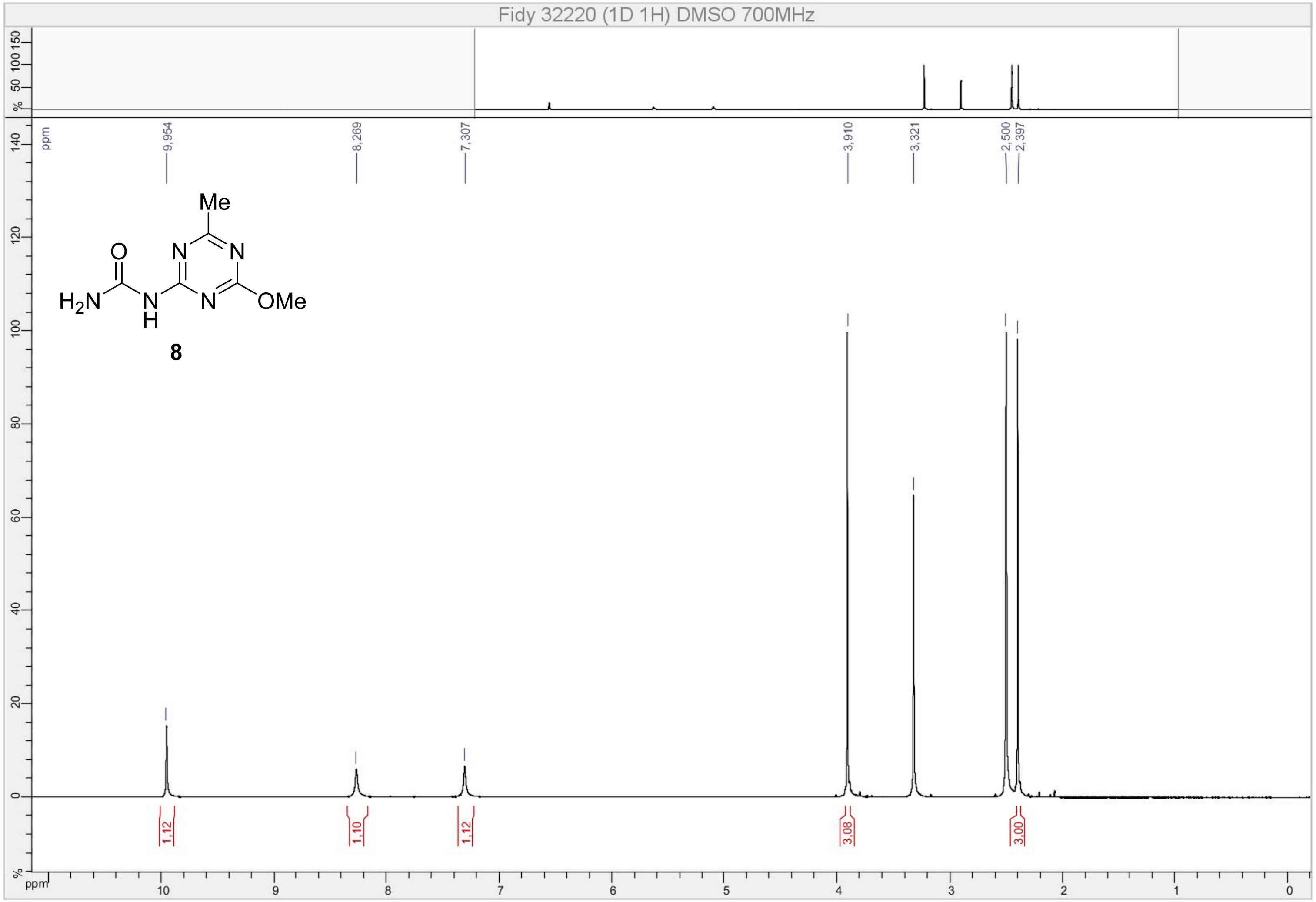

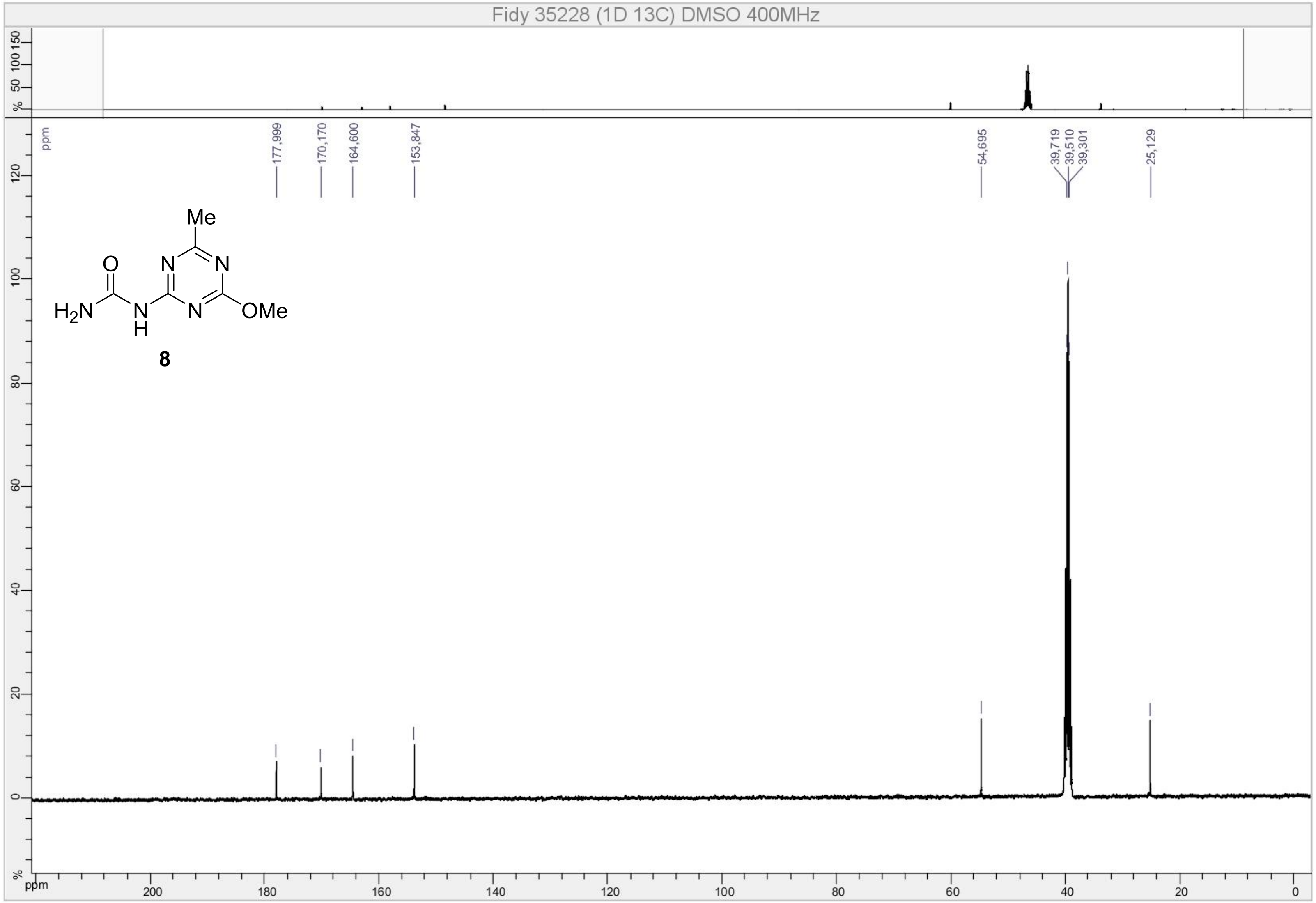
Fidy 35228 (1D 13C) DMSO 400MHz
177,999
170,170
164,600
153,847
54,695
39,719
39,510
39,301
25,129
Me
N
N
O
H2N
N
H
N
OMe
8
ppm
%

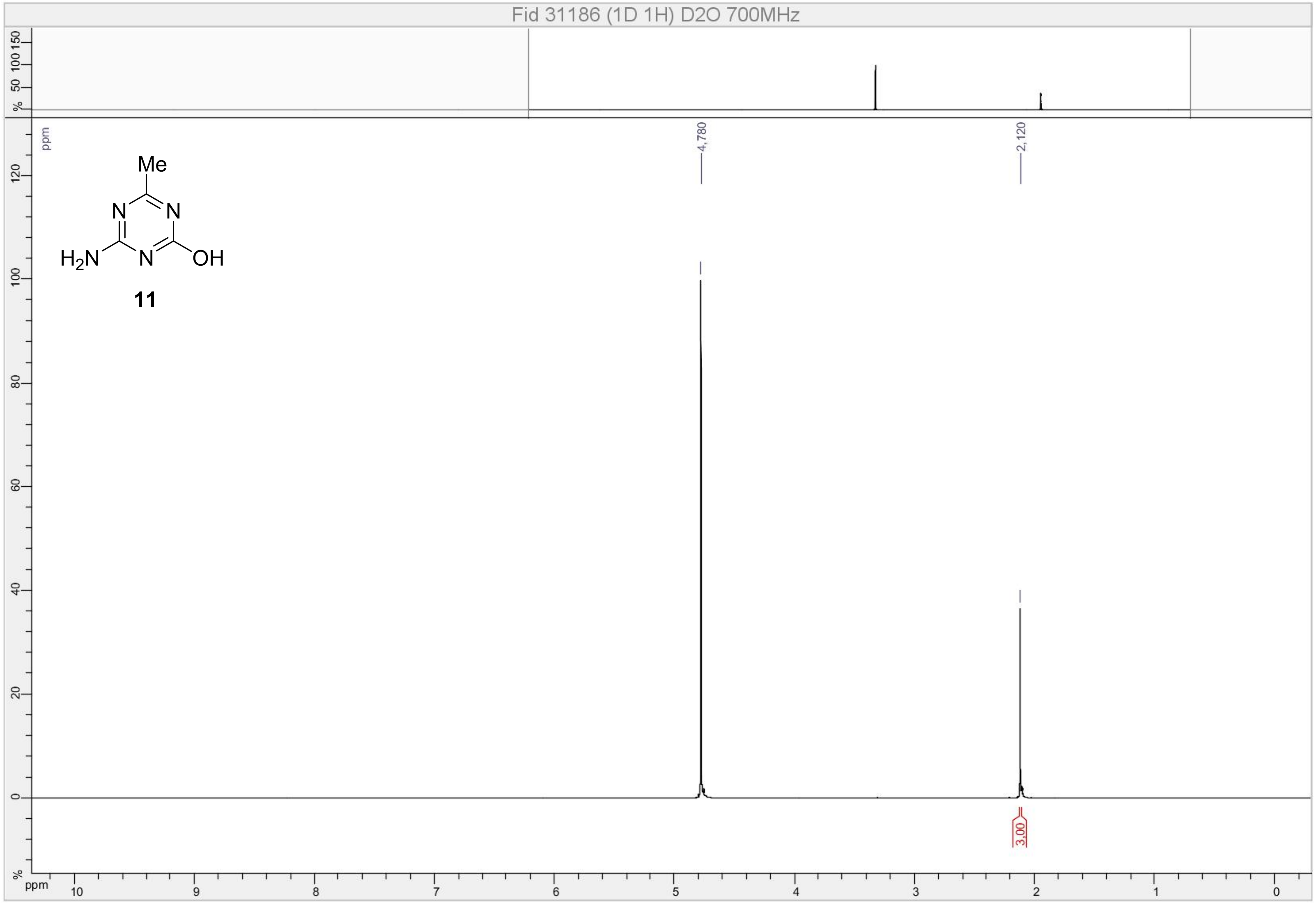
Fid 31186 (1D 1H) D2O 700MHz
Me
N
N
H2N
N
OH
11
4,780
2,120
3,00
ppm
%
10
9
8
7
6
5
4
3
2
1
0

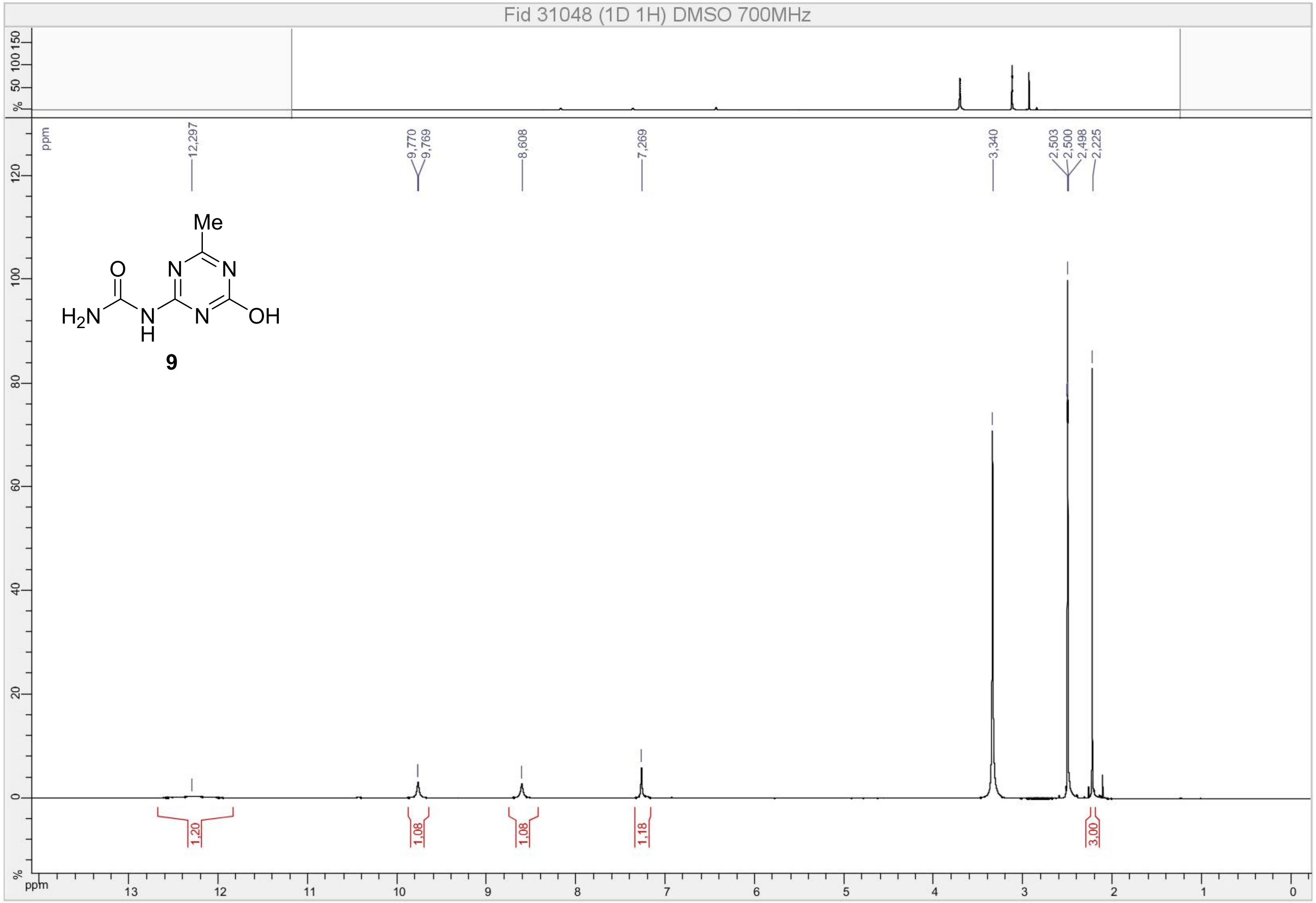
Fid 31048 (1D 1H) DMSO 700MHz
Me
O
N
N
H2N
N
H
N
OH
9
12,297
9,770
9,769
8,608
7,269
3,340
2,503
2,500
2,498
2,225
1,20
1,08
1,08
1,18
3,00
ppm

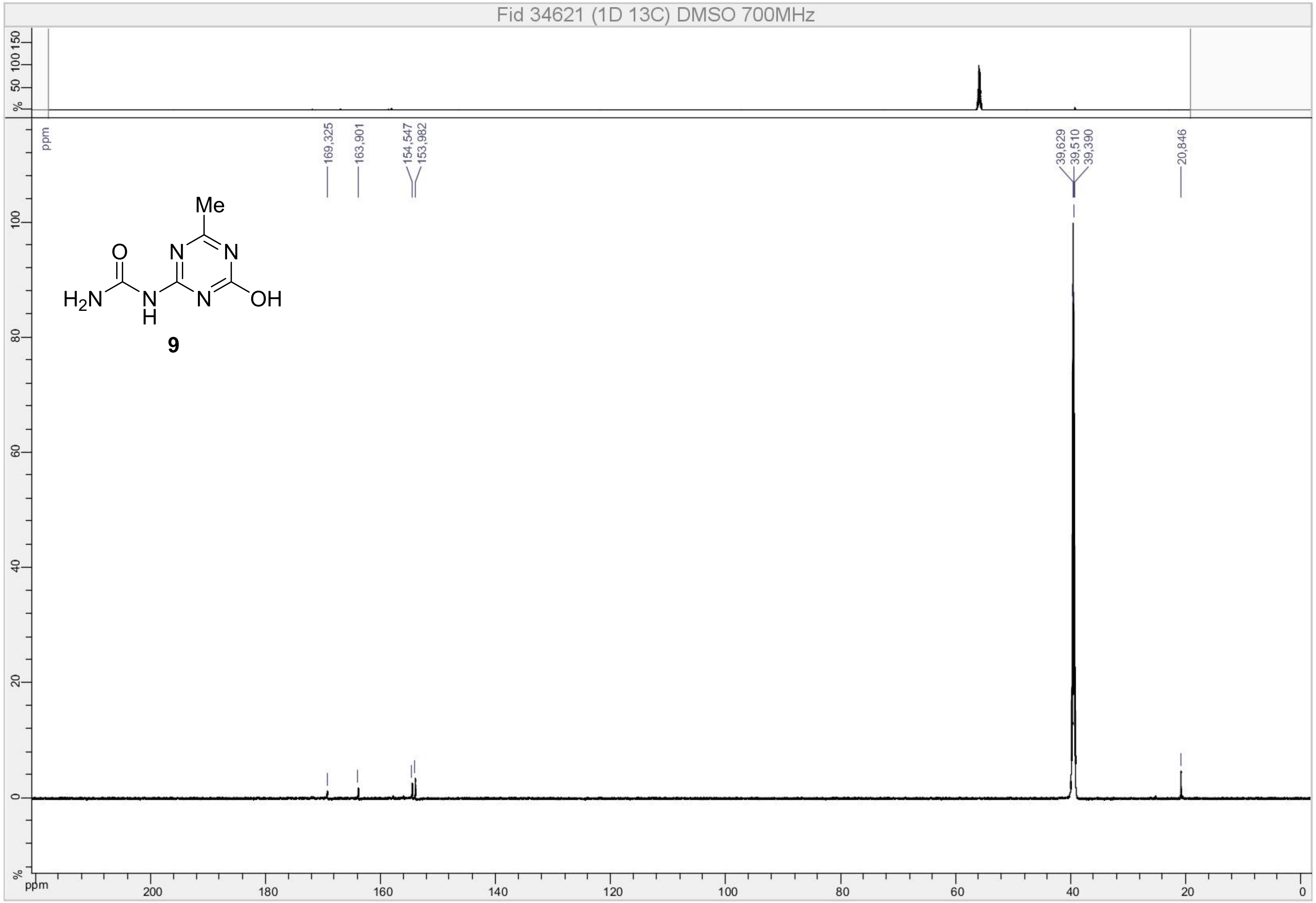
Fid 34621 (1D 13C) DMSO 700MHz
169,325
163,901
154,547
153,982
39,629
39,510
39,390
20,846
Me
O
N
N
N
H2N
N
H
OH
9
ppm
%
200
180
160
140
120
100
80
60
40
20
0

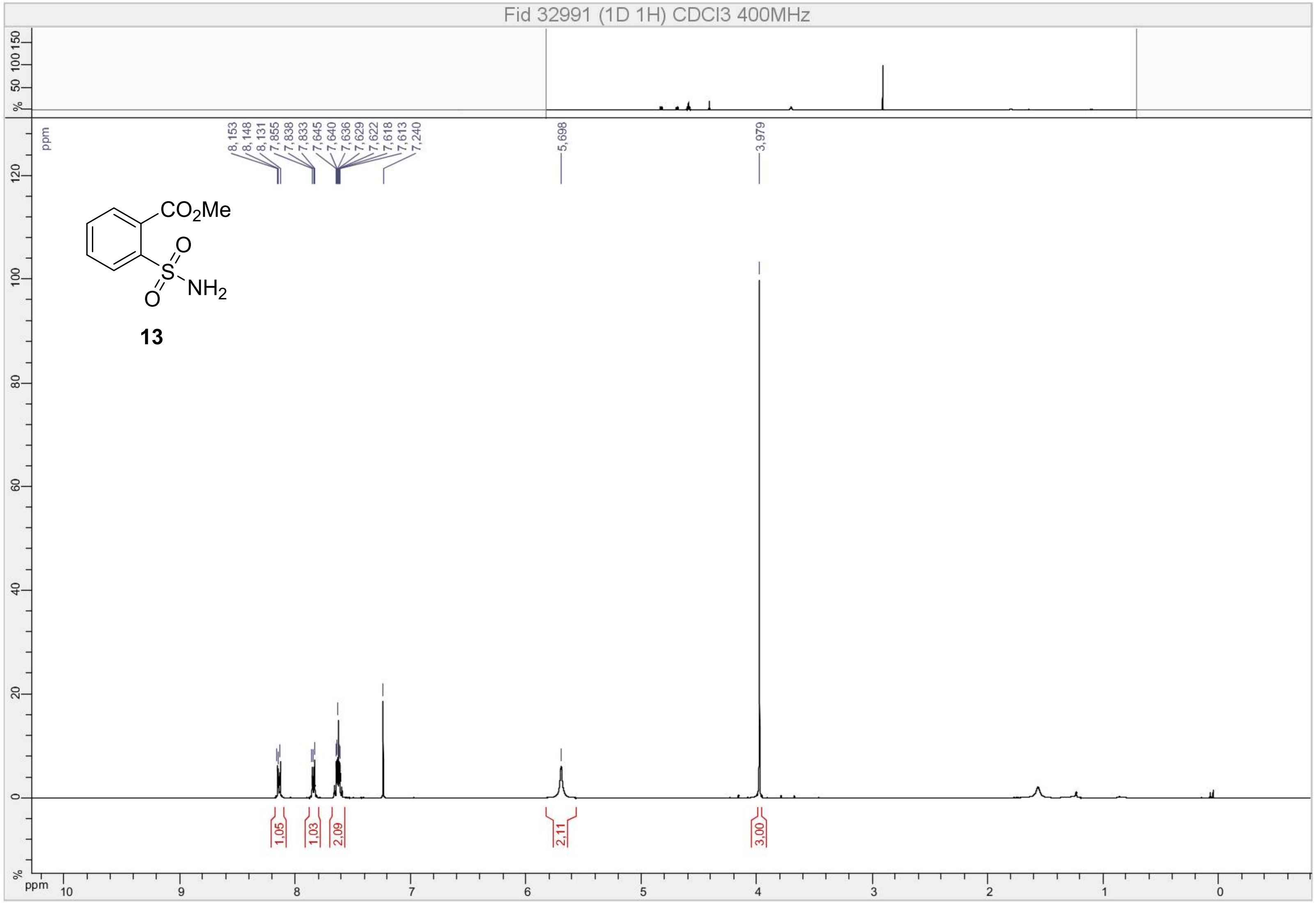
Fid 32991 (1D 1H) CDCl3 400MHz
8,153 8,148 8,131 7,855 7,838 7,833 7,645 7,640 7,636 7,629 7,622 7,618 7,613 7,240
5,698
3,979
CO2Me
S
O
O
NH2
13
1,05
1,03
2,09
2,11
3,00
ppm

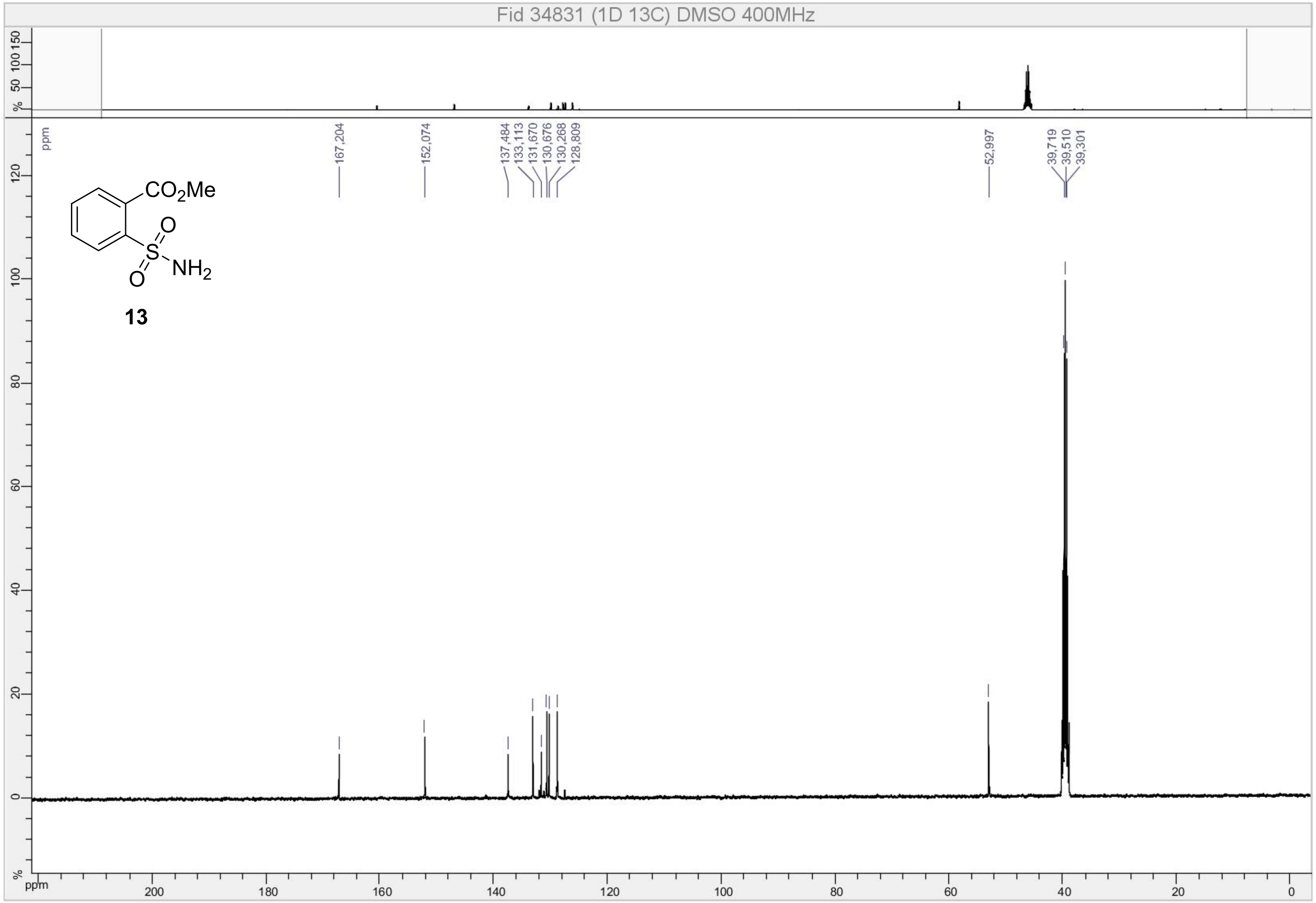
Fid 34831 (1D 13C) DMSO 400MHz
167,204
152,074
137,484
133,113
131,670
130,676
130,268
128,809
52,997
39,719
39,510
39,301
CO2Me
O
S
NH2
O
13
ppm
%

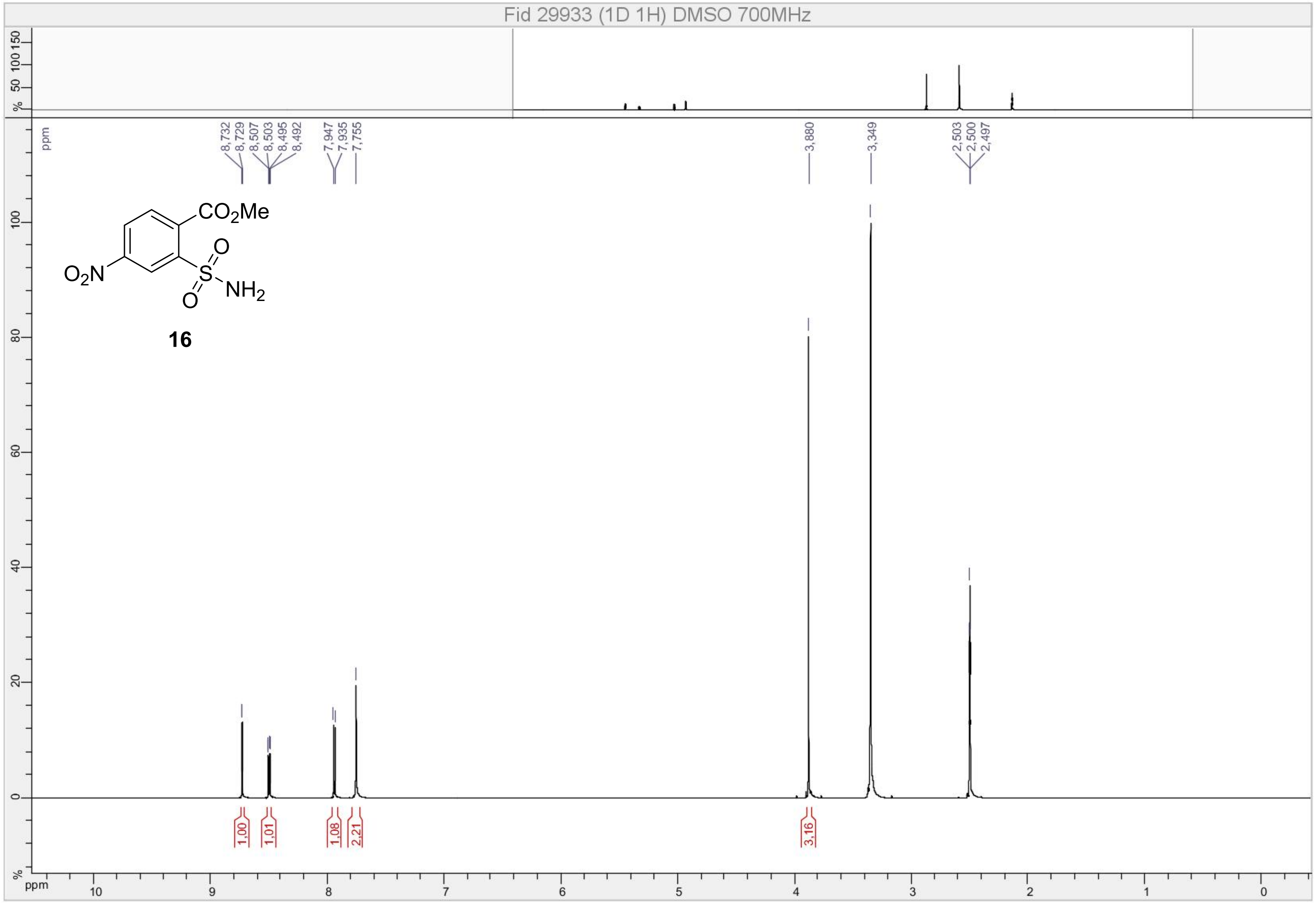
Fid 29933 (1D 1H) DMSO 700MHz
CO2Me
O2N
S
O
O
NH2
16
8,732
8,729
8,507
8,503
8,495
8,492
7,947
7,935
7,755
3,880
3,349
2,503
2,500
2,497
1,00
1,01
1,08
2,21
3,16
ppm
%

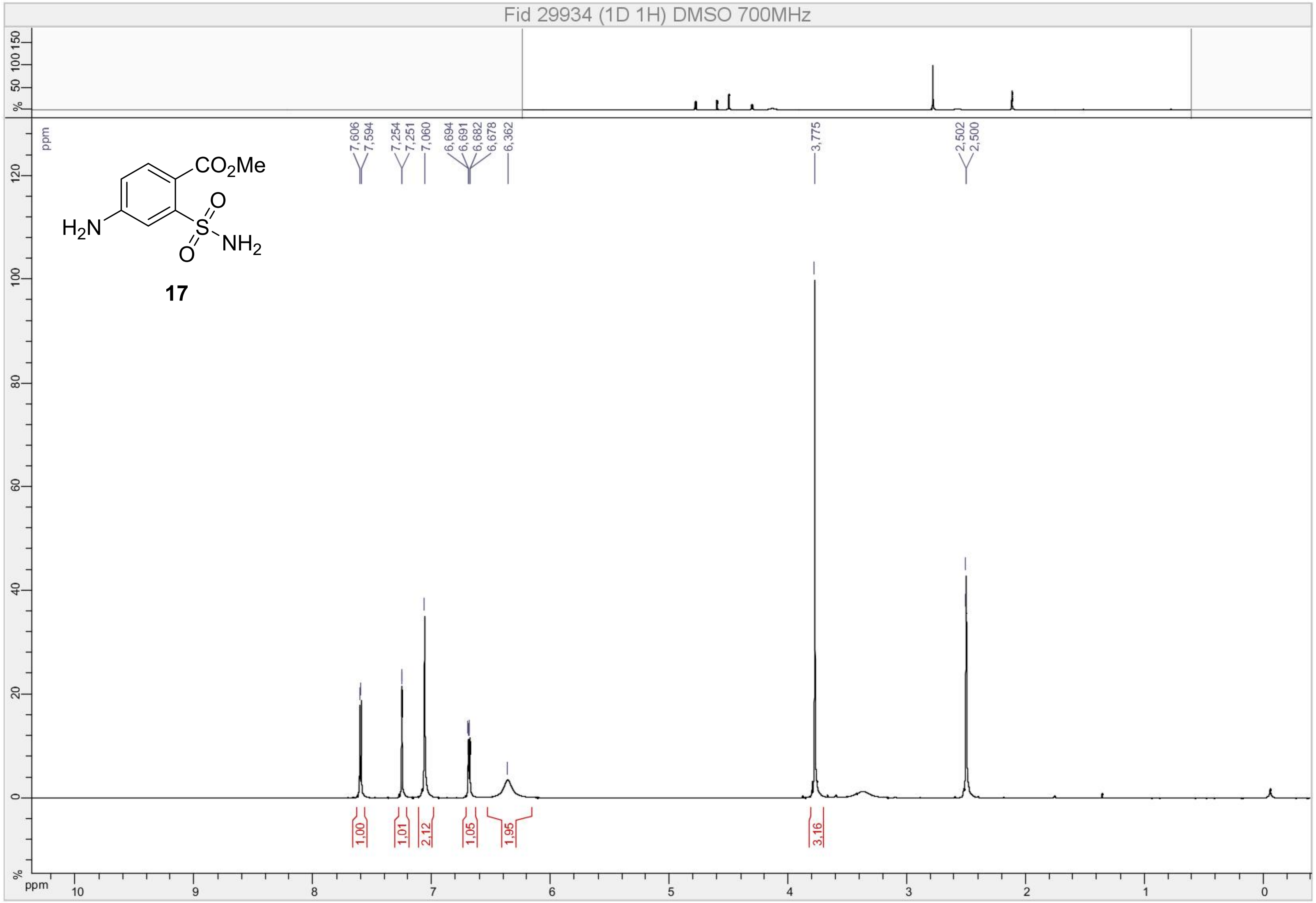

Fid 29934 (1D 1H) DMSO 700MHz
17

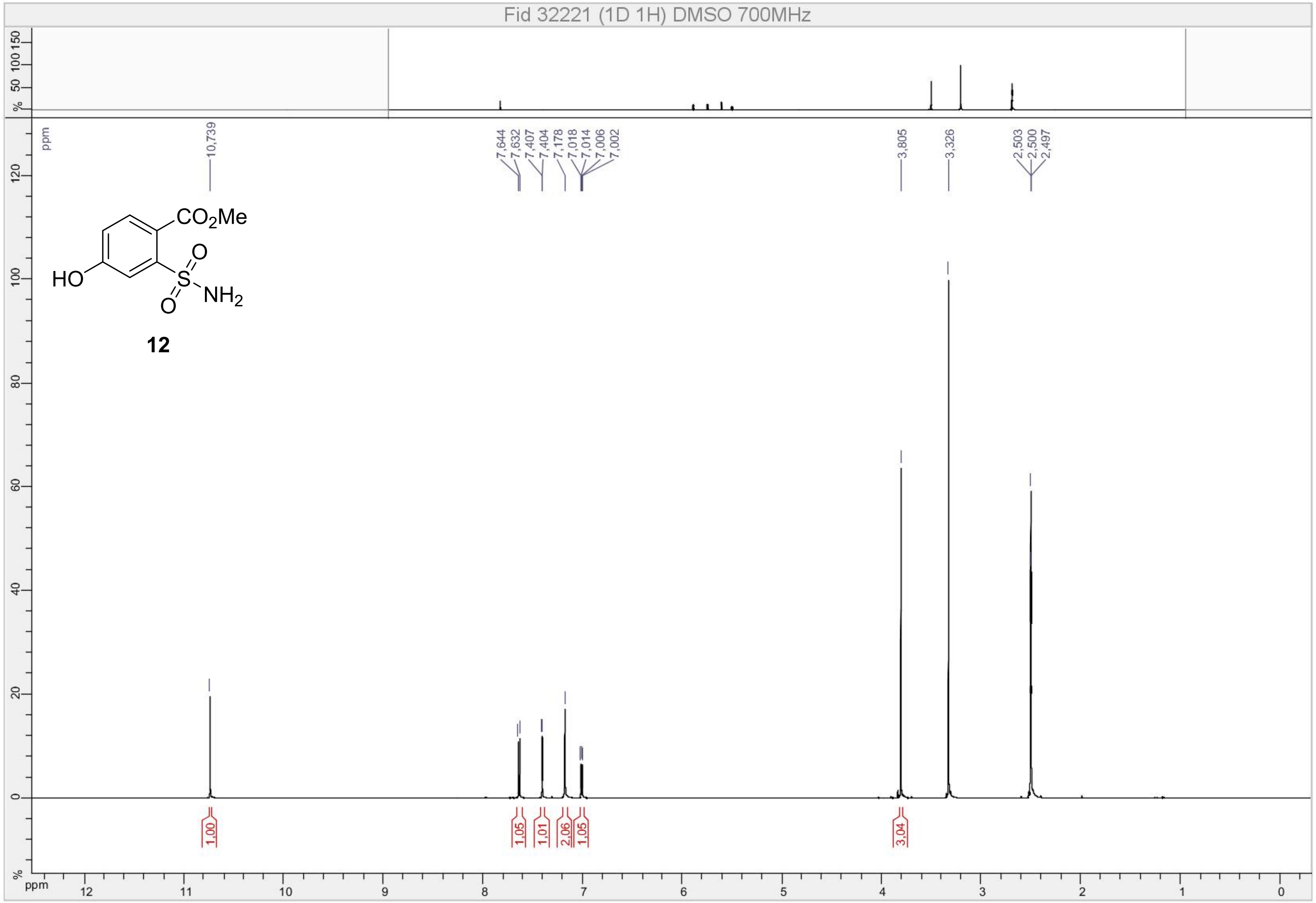
Fid 32221 (1D 1H) DMSO 700MHz
10,739
7,644
7,632
7,407
7,404
7,178
7,018
7,014
7,006
7,002
3,805
3,326
2,503
2,500
2,497
CO2Me
HO
O
S
NH2
O
12
1,00
1,05
1,01
2,06
1,05
3,04
ppm
%

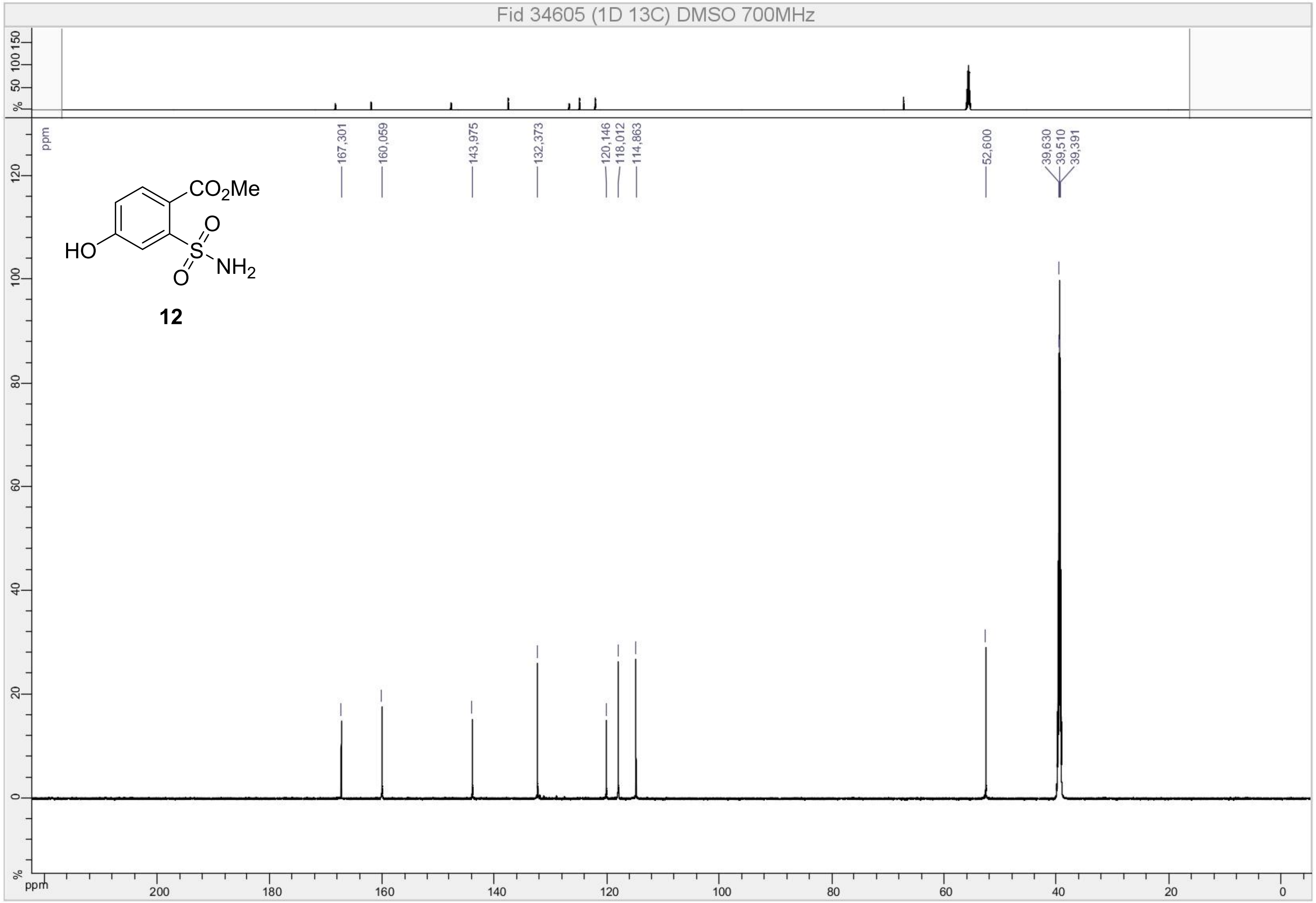
Fid 34605 (1D 13C) DMSO 700MHz
167,301
160,059
143,975
132,373
120,146
118,012
114,863
52,600
39,630
39,510
39,391
CO2Me
HO
O
S
NH2
O
12
ppm
%
200
180
160
140
120
100
80
60
40
20
0

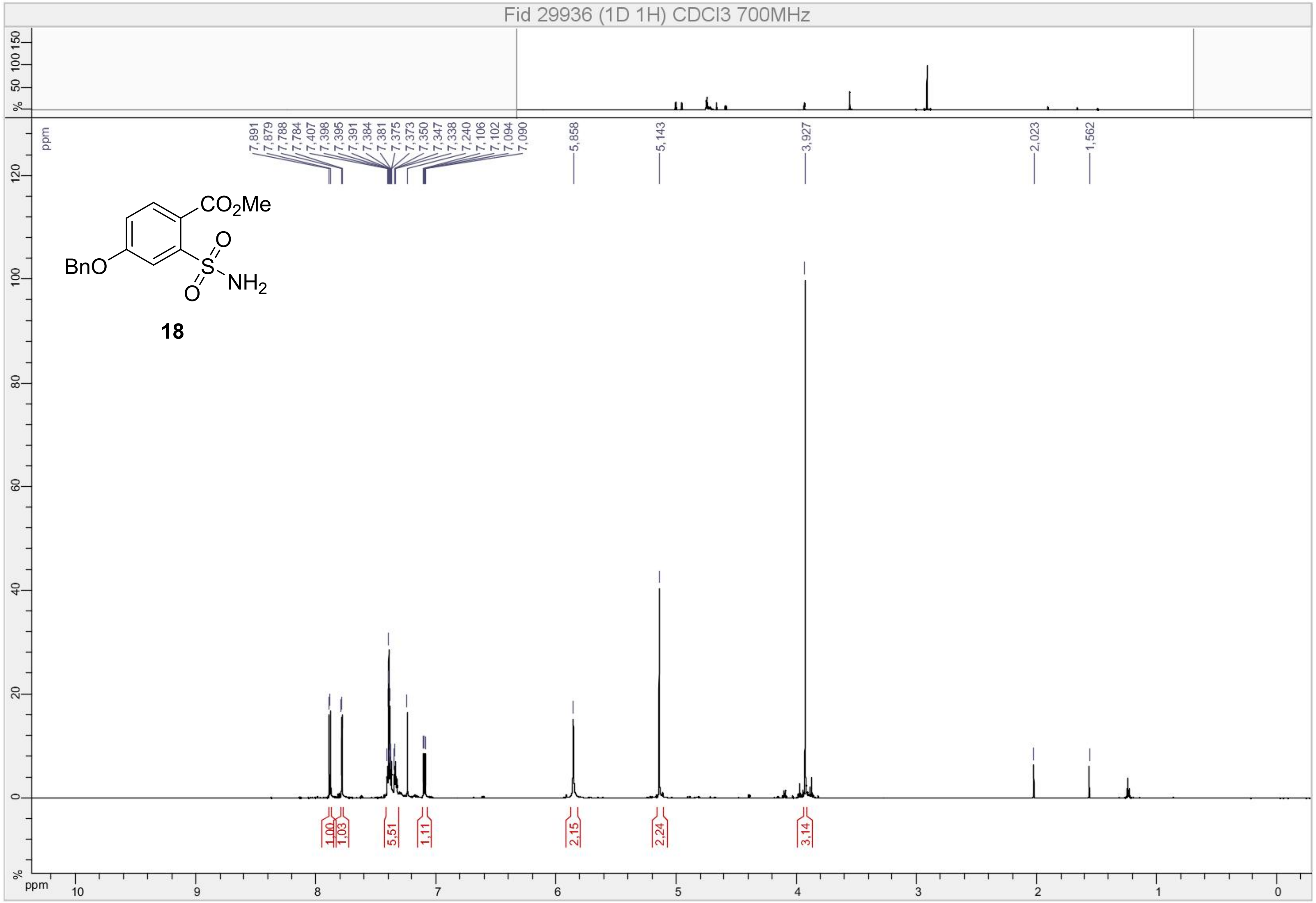

Fid 29936 (1D 1H) CDCl3 700MHz
7,891
7,879
7,788
7,784
7,407
7,398
7,395
7,391
7,384
7,381
7,375
7,373
7,350
7,347
7,338
7,240
7,106
7,102
7,094
7,090
5,858
5,143
3,927
2,023
1,562
1,00
1,03
5,51
1,11
2,15
2,24
3,14
ppm
CO2Me
BnO
O
S
NH2
O

18

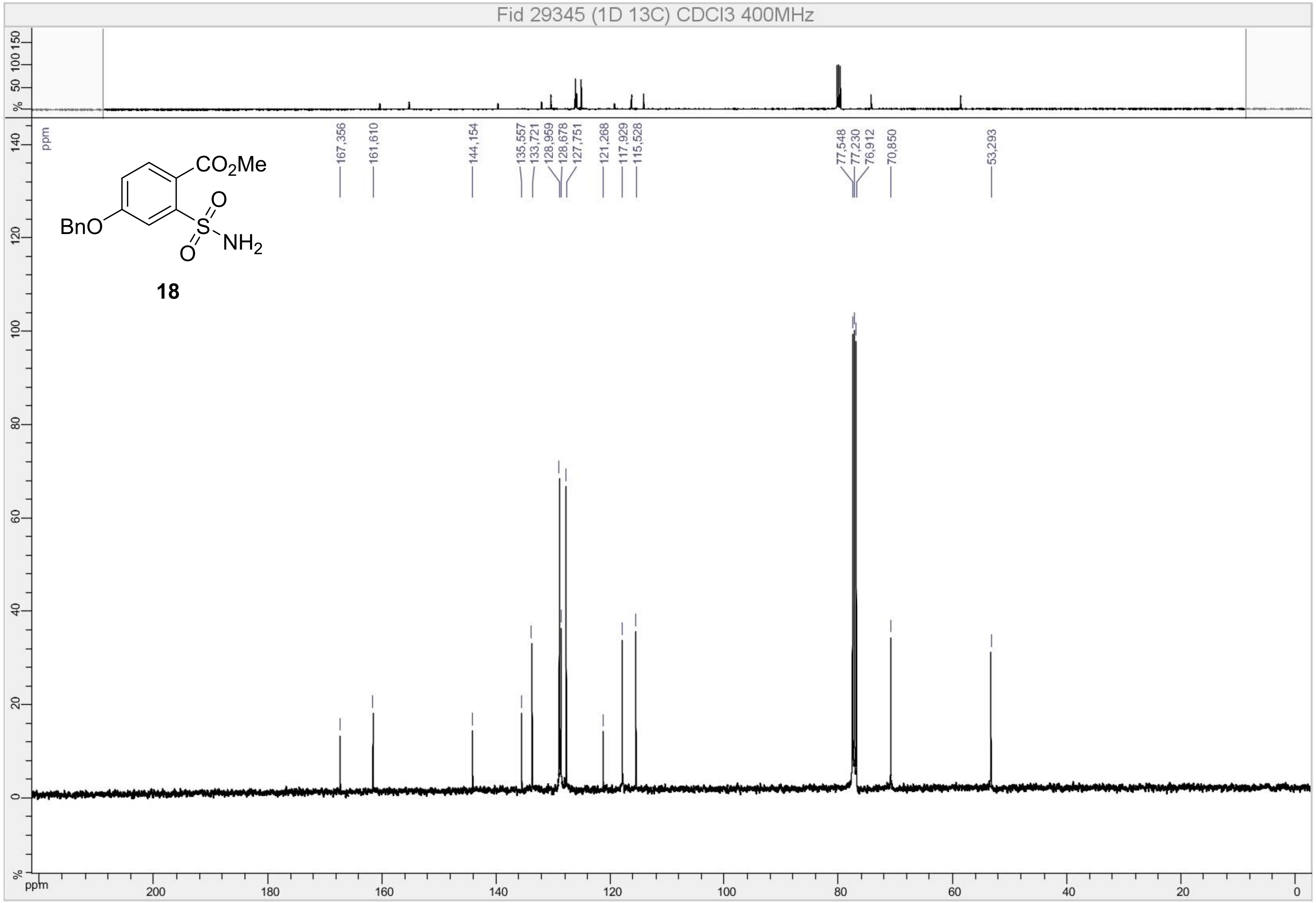
Fid 29345 (1D 13C) CDCl3 400MHz
CO2Me
BnO
S
O
O
NH2
18
167,356
161,610
144,154
135,557
133,721
128,959
128,678
127,751
121,268
117,929
115,528
77,548
77,230
76,912
70,850
53,293
ppm
200
180
160
140
120
100
80
60
40
20
0

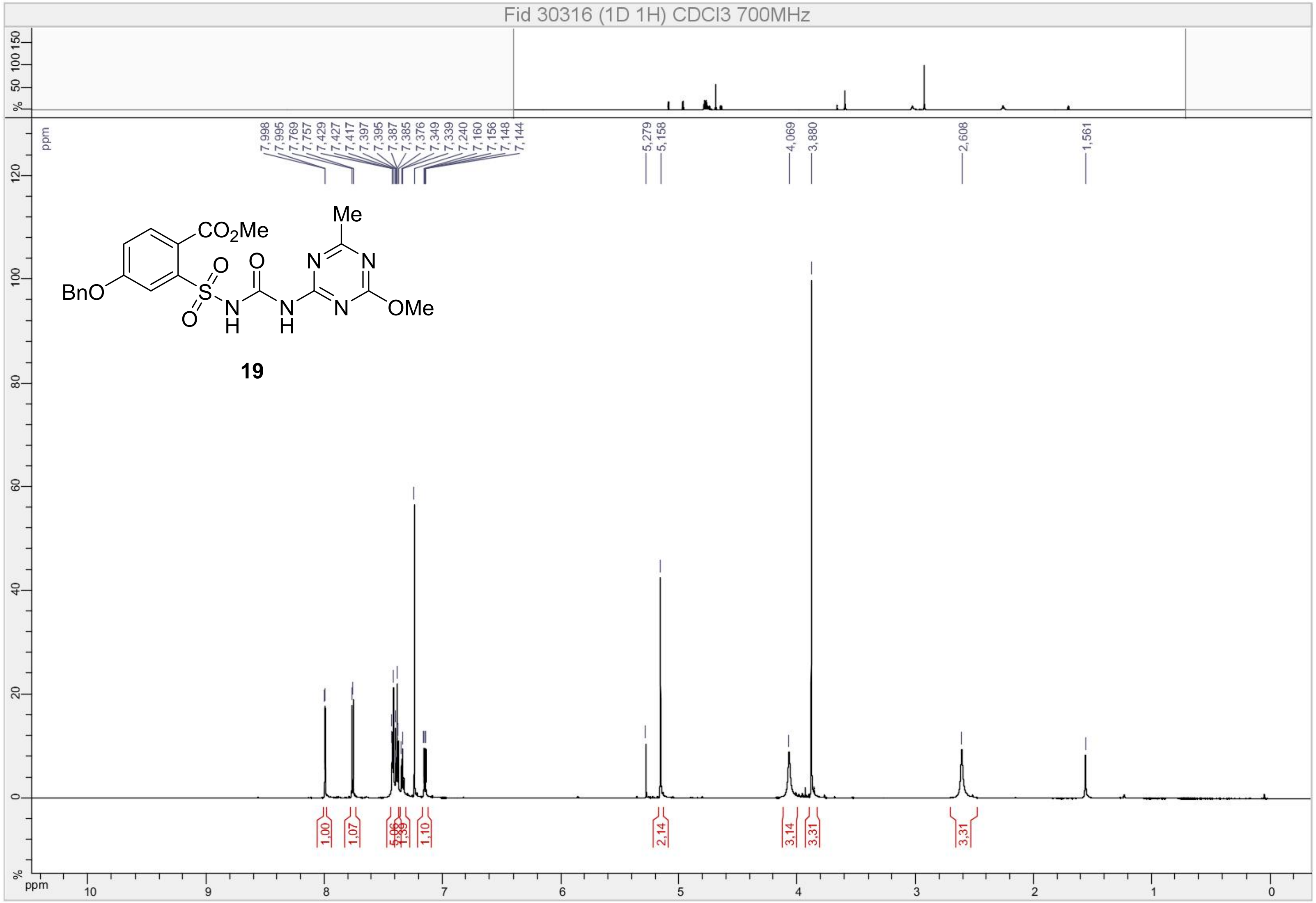

Fid 30316 (1D 1H) CDCl3 700MHz
7,998
7,995
7,769
7,757
7,429
7,427
7,417
7,397
7,395
7,387
7,385
7,376
7,349
7,339
7,240
7,160
7,156
7,148
7,144
5,279
5,158
4,069
3,880
2,608
1,561
CO2Me
Me
BnO
19
OMe
1,00
1,07
1,10
2,14
3,14
3,31
3,31
ppm

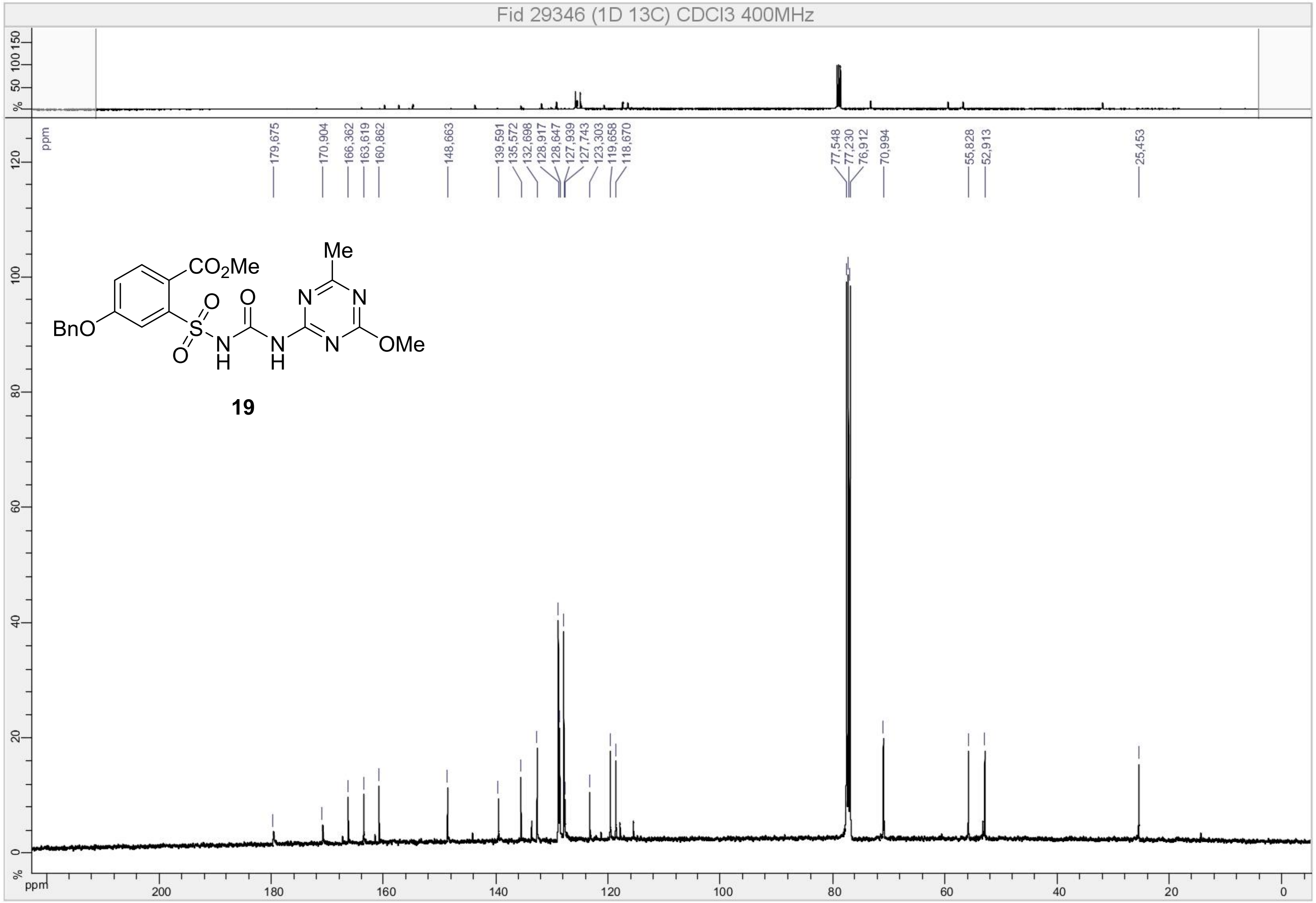

Fid 29346 (1D 13C) CDCl3 400MHz
ppm
179,675
170,904
166,362
163,619
160,862
148,663
139,591
135,572
132,698
128,917
128,647
127,939
127,743
123,303
119,658
118,670
77,548
77,230
76,912
70,994
55,828
52,913
25,453
CO2Me
BnO
Me
OMe
19

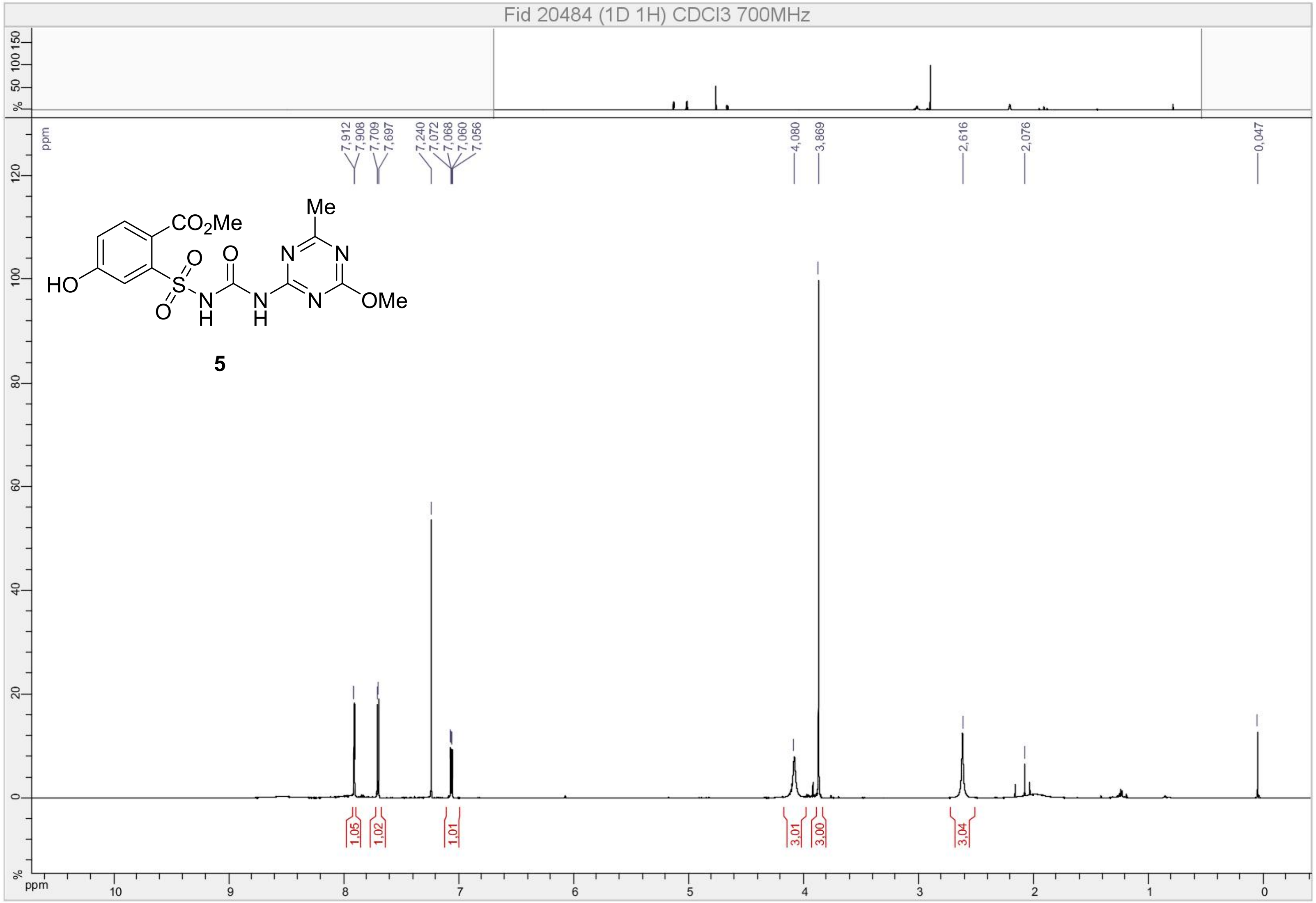
Fid 20484 (1D 1H) CDCl3 700MHz
7,912
7,908
7,709
7,697
7,240
7,072
7,068
7,060
7,056
4,080
3,869
2,616
2,076
0,047
1,05
1,02
1,01
3,01
3,00
3,04
CO2Me
Me
HO
OMe
5
ppm

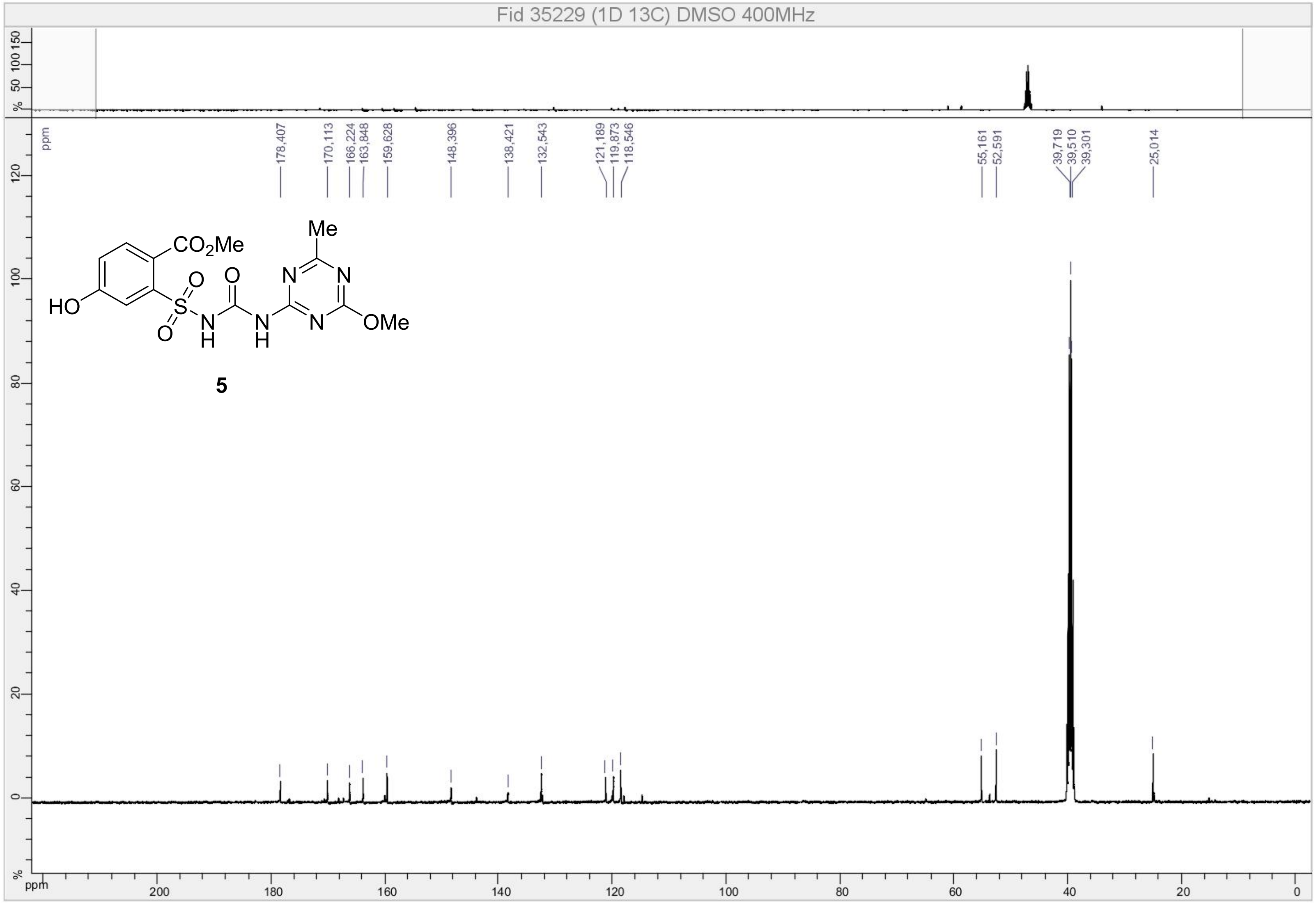
Fid 35229 (1D 13C) DMSO 400MHz
178,407
170,113
166,224
163,848
159,628
148,396
138,421
132,543
121,189
119,873
118,546
55,161
52,591
39,719
39,510
39,301
25,014
ppm
CO2Me
Me
HO
OMe
5

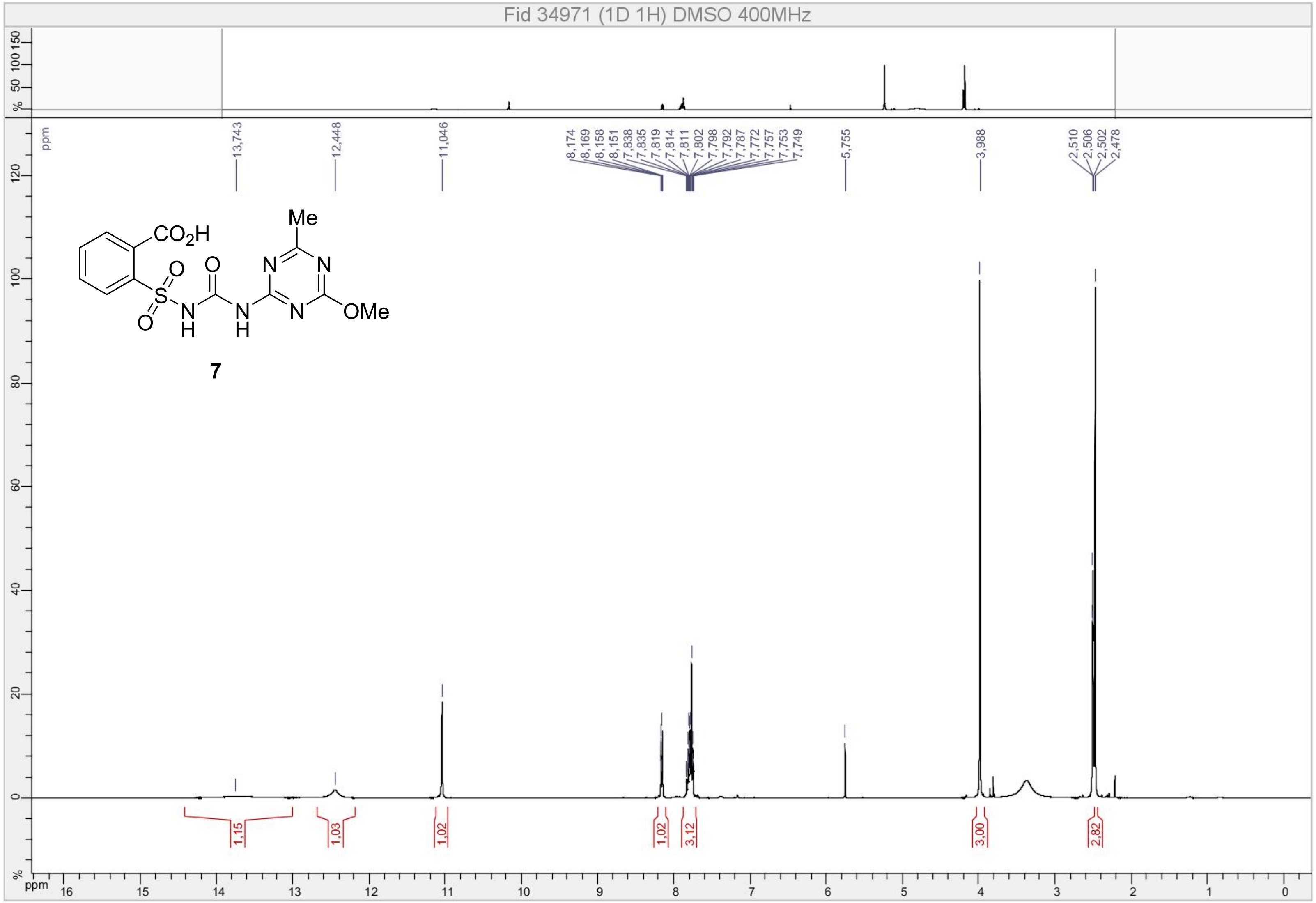

Fid 34971 (1D 1H) DMSO 400MHz
CO2H
Me
O
O
O
N
N
S
N
N
N
OMe
H
H
N
7
13,743
12,448
11,046
8,174
8,169
8,158
8,151
7,838
7,835
7,819
7,814
7,811
7,802
7,798
7,792
7,787
7,772
7,757
7,753
7,749
5,755
3,988
2,510
2,506
2,502
2,478
1,15
1,03
1,02
1,02
3,12
3,00
2,82
ppm

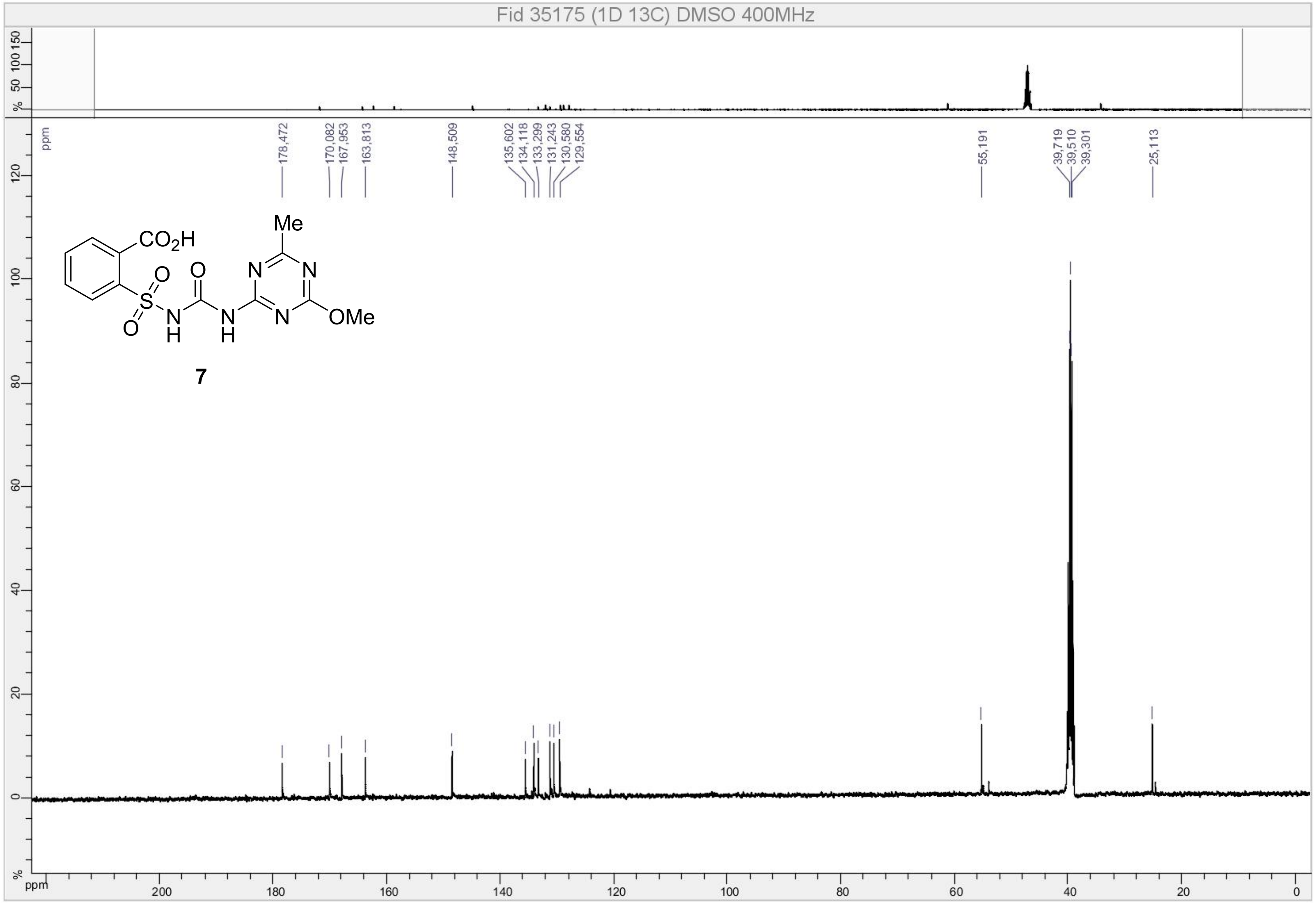
Fid 35175 (1D 13C) DMSO 400MHz
178,472
170,082
167,953
163,813
148,509
135,602
134,118
133,299
131,243
130,580
129,554
55,191
39,719
39,510
39,301
25,113
CO2H
Me
OMe
7
ppm
%

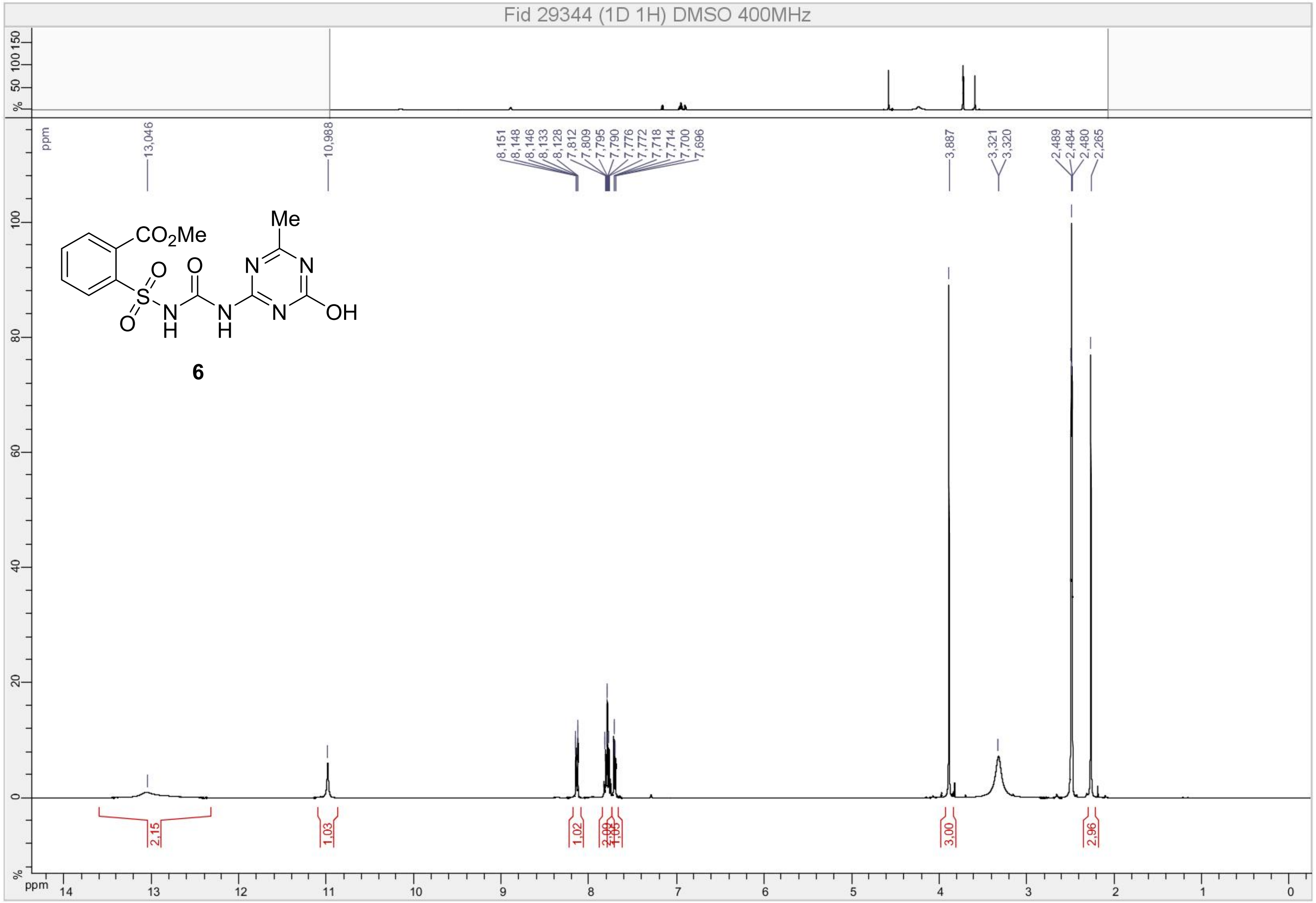

Fid 29344 (1D 1H) DMSO 400MHz
13,046
10,988
8,151 8,148 8,146 8,133 8,128 7,812 7,809 7,795 7,790 7,776 7,772 7,718 7,714 7,700 7,696
3,887
3,321 3,320
2,489 2,484 2,480 2,265
CO2Me
Me
6
2,15
1,03
1,02
3,00
2,96
ppm

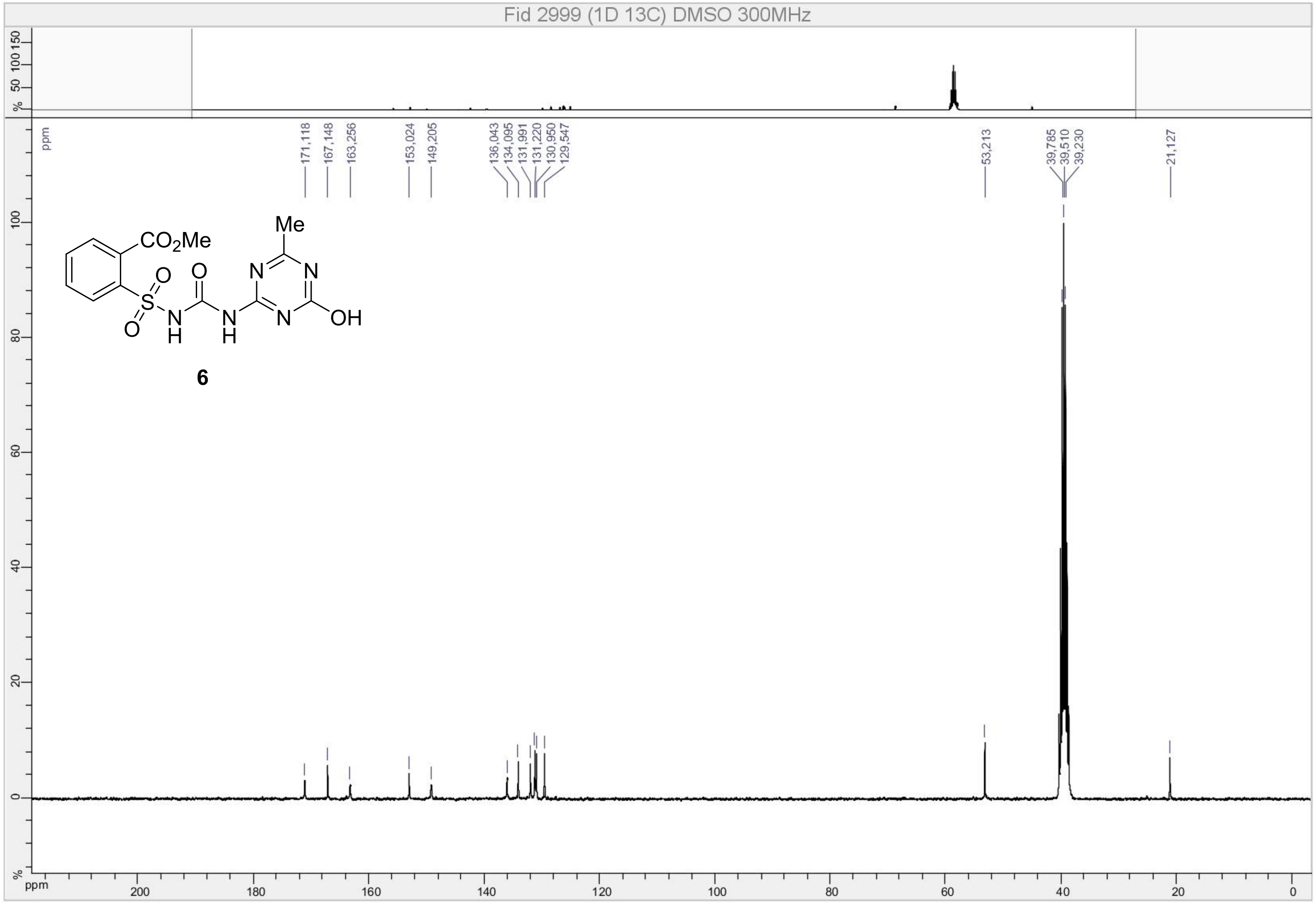
Fid 2999 (1D 13C) DMSO 300MHz
171,118
167,148
163,256
153,024
149,205
136,043
134,095
131,991
131,220
130,950
129,547
53,213
39,785
39,510
39,230
21,127
ppm
%
CO2Me
Me
N
O
OH
H
S
6

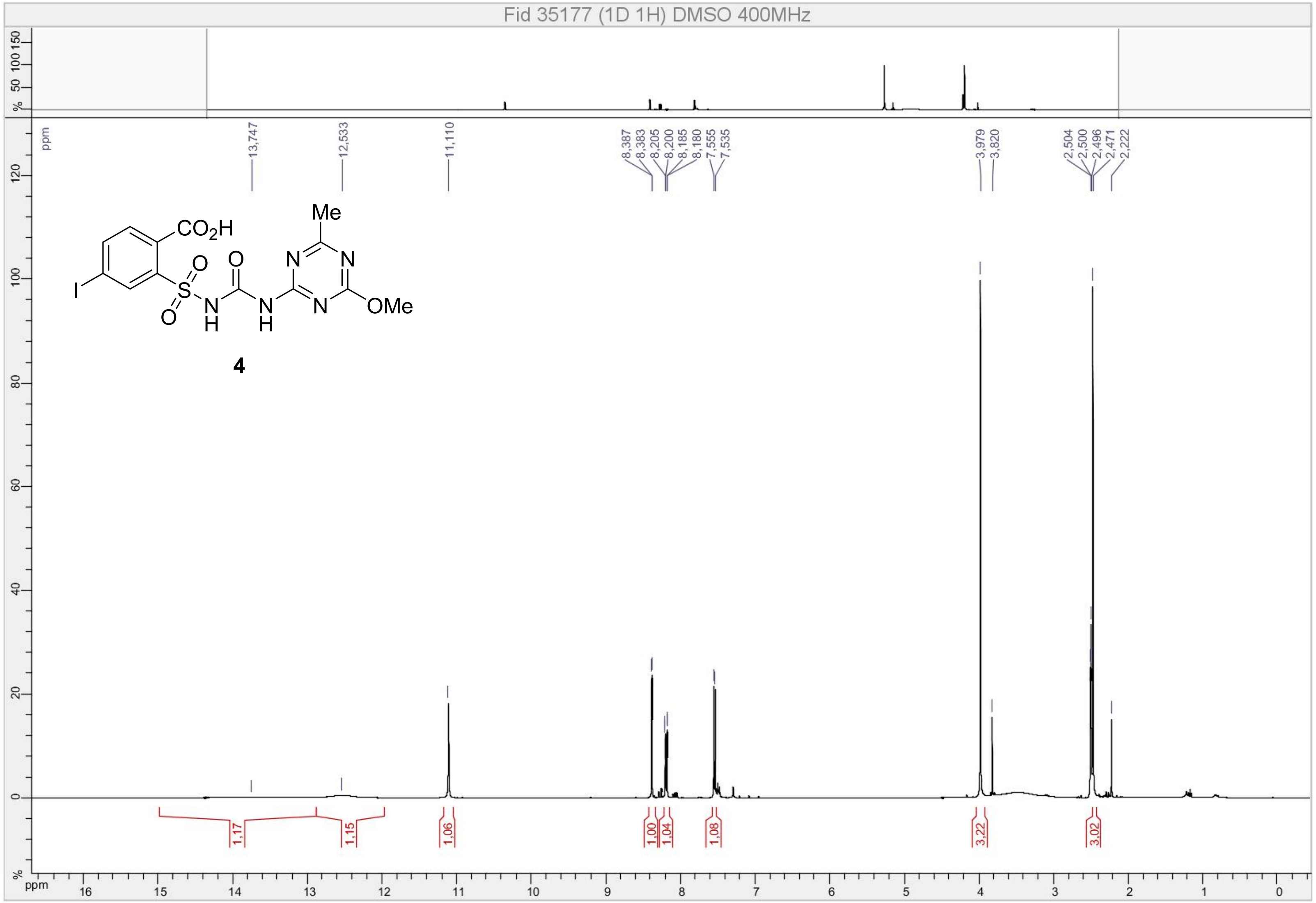
Fid 35177 (1D 1H) DMSO 400MHz
13,747
12,533
11,110
8,387
8,383
8,205
8,200
8,185
8,180
7,555
7,535
3,979
3,820
2,504
2,500
2,496
2,471
2,222
CO2H
Me
N
N
O
O
S
O
N
H
N
H
N
OMe
I
4
1,17
1,15
1,06
1,00
1,04
1,08
3,22
3,02
ppm

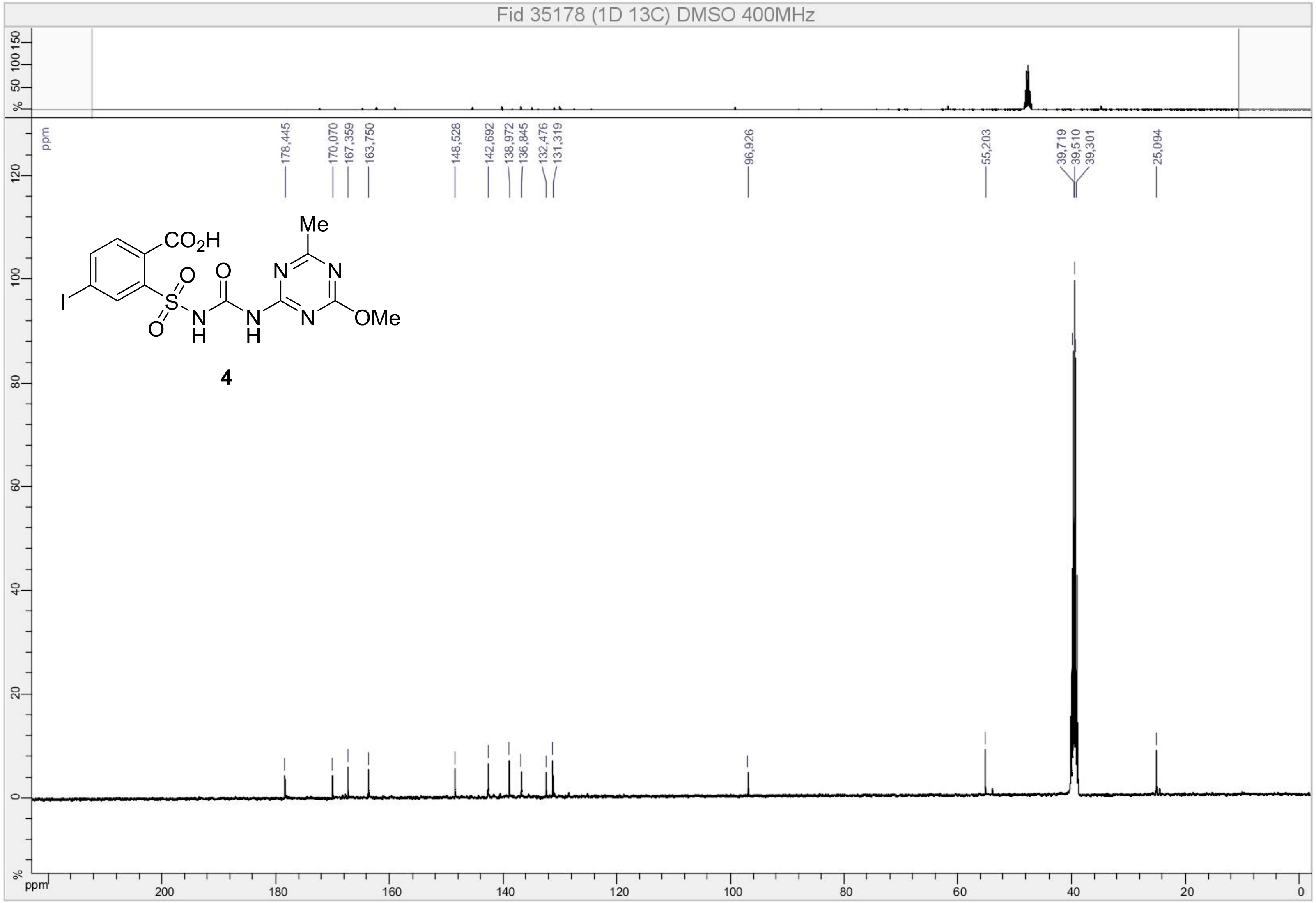
Fid 35178 (1D 13C) DMSO 400MHz
178,445
170,070
167,359
163,750
148,528
142,692
138,972
136,845
132,476
131,319
96,926
55,203
39,719
39,510
39,301
25,094
ppm
%
CO2H
Me
N
N
O
O
I
S
N
N
N
OMe
O
H
H
4

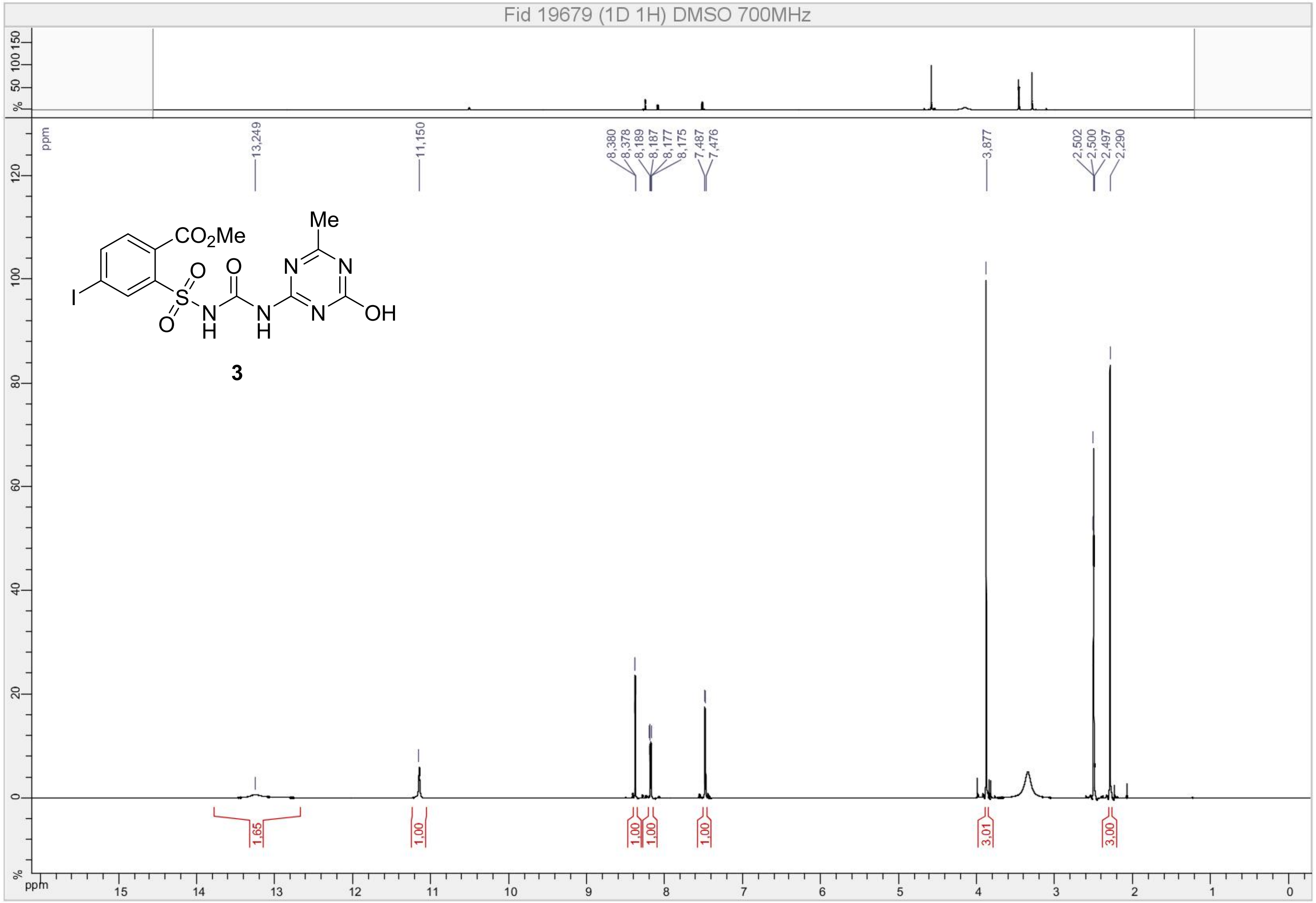
Fid 19679 (1D 1H) DMSO 700MHz
CO2Me
Me
O
O
N
N
I
S
N
N
N
OH
O
H
H
3
13,249
11,150
8,380
8,378
8,189
8,187
8,177
8,175
7,487
7,476
3,877
2,502
2,500
2,497
2,290
1,65
1,00
1,00
1,00
1,00
3,01
3,00
ppm

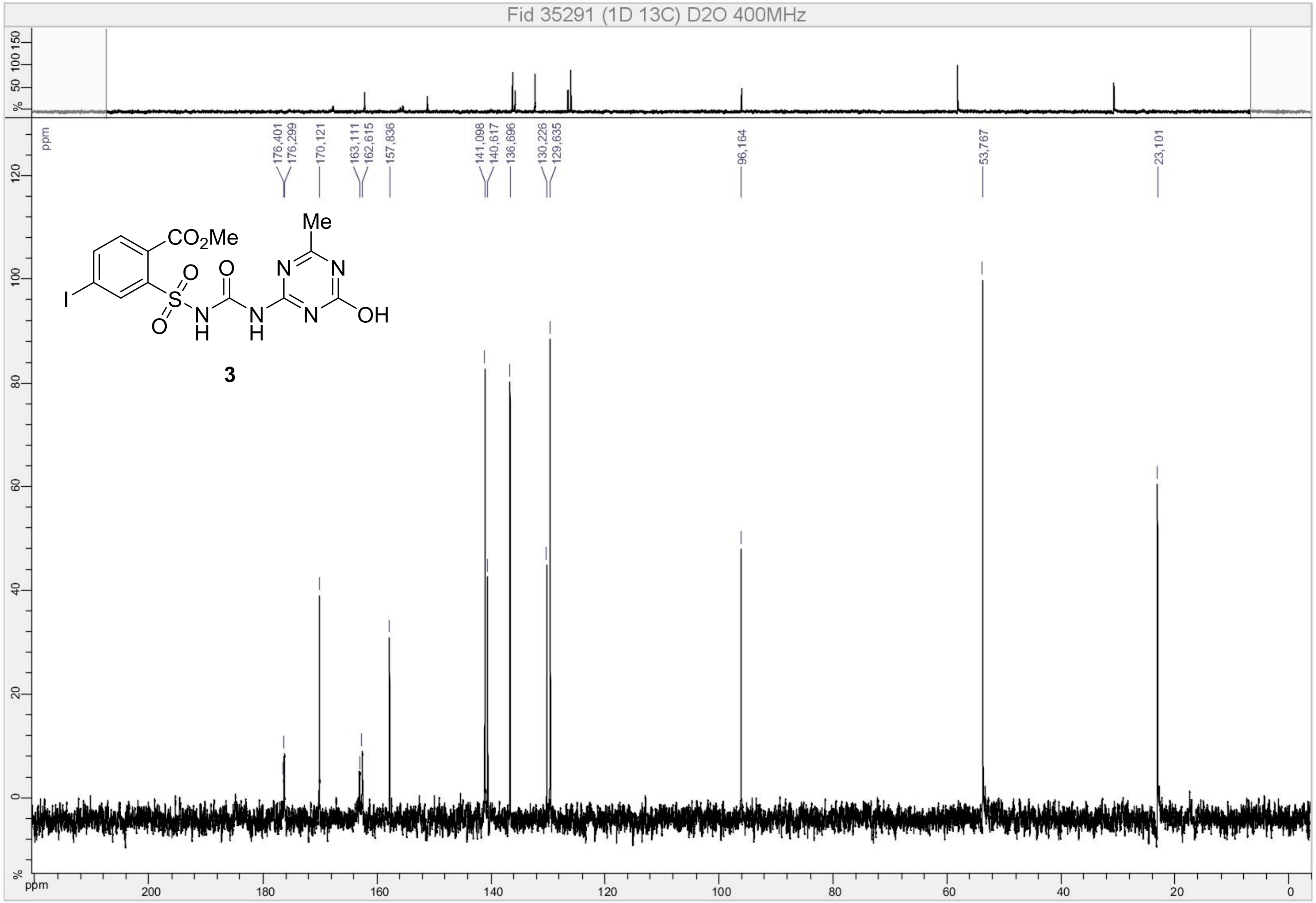

Fid 35291 (1D 13C) D2O 400MHz
176,401
176,299
170,121
163,111
162,615
157,836
141,098
140,617
136,696
130,226
129,635
96,164
53,767
23,101
ppm
%
CO2Me
Me
I
3
